# Crystal structure of chain silicate Cs_3_LuSi_3_O_9_

**DOI:** 10.1107/S2056989021011439

**Published:** 2021-11-04

**Authors:** Hiromitsu Kimura, Hisanori Yamane

**Affiliations:** a Inorganic Materials Laboratory, Science & Innovation Center, Mitsubishi Chemical Corporation, 1000 Kamoshida-cho, Aoba-ku, Yokohama-shi, Kanagawa, 227-8502, Japan; b Institute of Multidisciplinary Research for Advanced Material, Tohoku University, 2-1-1 Katahira, Aoba-ku, Sendai, Miyagi, 980-8577, Japan

**Keywords:** caesium lutetium(III) silicate, crystal structure, single-chain silicate

## Abstract

A caesium lutetium(III) silicate, Cs_3_LuSi_3_O_9_, was synthesized by heating a pelletized mixture of Cs_2_CO_3_, Lu_2_O_3_ and SiO_2_ at 1273 K. Single crystals of the title compound were grown in a melted area of the pellet. Cs_3_LuSi_3_O_9_ is a single-chain silicate (ortho­rhom­bic space group *Pna*2_1_) with a chain periodicity of six and is isostructural with Cs_3_ *RE* ^III^Ge_3_O_9_ (*RE* = Pr, Nd and Sm–Yb). The two symmetry-dependent [Si_6_O_18_]^12−^ chains in the unit cell lie parallel to the [011] direction. The Lu^3+^ ions are octa­hedrally coordinated by O atoms of the silicate chains, generating a three-dimensional framework. Cs^+^ ions are located in the voids in the framework.

## Abstract 2

Caesium lutetium(III) silicate, Cs_3_LuSi_3_O_9_, is a sechser single-chain silicate in which [Si_6_O_18_]^12−^ chains are linked together *via* octa­hedrally coordinated Lu^3+^ ions, generating a three-dimensional framework structure. Cs^+^ ions reside in the voids in the framework.

## Chemical context

A lutetium(III) silicate, Lu_2_SiO_5_, containing the highest atomic weight rare-earth element, has been studied as the host crystal of a scintillator for radiation detection (Dorenbos *et al.*, 1994 ▸; Melcher & Schweitzer, 1992 ▸). Our research group recently reported the syntheses and crystal structures of a new oxide and oxynitride containing Lu; namely, Lu_4_Al_2_O_9_ (Simura & Yamane, 2020*a* ▸) and Ba_0.9_Ce_0.1_LuAl_0.2_Si_3.8_N_6.9_O_0.1_ (Simura & Yamane, 2020*b* ▸). In the present study, a new quaternary single-chain silicate, Cs_3_LuSi_3_O_9_, was found during an exploratory study of the Cs–Lu–Si–O system. In other quaternary silicate systems containing alkali-metal (*A*) and rare-earth (*RE*) elements, a number of single-chain silicates have recently been reported: LiScSi_2_O_6_ (space groups *C*2/*c*, *P*2_1_/*c*; Arlt & Angel, 2000 ▸; Redhammer & Roth, 2004 ▸), NaYSi_2_O_6_ (*P*2_1_/*c*; Többens *et al.*, 2005 ▸), NaTbSi_2_O_6_ (*P*2_1_ */c*; Schäfer *et al.*, 2012 ▸), Na_3_YSi_3_O_9_ (*P*2_1_2_1_2_1_; Maksimov *et al.*, 1980 ▸), Na_3_ *RE*Si_3_O_9_ (*RE* = Y, Dy, Gd, *P*2_1_2_1_2_1_; Shannon *et al.*, 1980 ▸), Na_3_ *RE*Si_3_O_9_ (*RE* = Y, Er, *P*2_1_2_1_2_1_; Ananias *et al.*, 2002 ▸), Na_3_TmSi_3_O_9_ (*P*2_1_2_1_2_1_; Kahlenberg *et al.*, 2015 ▸), K_3_HoSi_3_O_9_ (*Pm*2_1_ *n*; Ponomarev *et al.*, 1988 ▸), K_3_ *RE*Si_3_O_9_ (*RE* = Ho, Tm, Lu, *Pm*2_1_ *n*; Filipenko *et al.*, 1988 ▸), K_3_TbSi_3_O_9_ (*Pm*2_1_ *n*; Kostova *et al.*, 2007 ▸), and K_2.9_Rb_0.1_ErSi_3_O_9_ (*P*1; Wierzbicka-Wieczorek *et al.*, 2010 ▸). Na^+^ ion conduction and photoluminescence have been investigated on doping Eu, Tb and Tm into Na_3_YSi_3_O_9_ (Shannon *et al.*, 1980 ▸; Kim *et al.*, 1985 ▸; Banks & Kim, 1985 ▸; Ananias *et al.*, 2006 ▸; Zhang *et al.*, 2008*a* ▸,*b* ▸). Photoluminescence properties have also been characterized for Na_3_ErSi_3_O_9_ (Ananias *et al.*, 2002 ▸) and K_3_ *RE* Si_3_O_9_ (*RE* = Y, Eu, Tb; Kostova *et al.*, 2007 ▸). A series of germanates with a new structure type, Cs_3_ *RE* ^III^Ge_3_O_9_ (*RE* = Pr, Nd and Sm-Yb, space group *Pna*2_1_) have recently been reported (Morrison *et al.*, 2019 ▸). In the present study, we report the synthesis and structural characterization of Cs_3_LuSi_3_O_9_, the first silicate found to be isostructural with the germanates.

## Structural commentary

**Figure 1:**
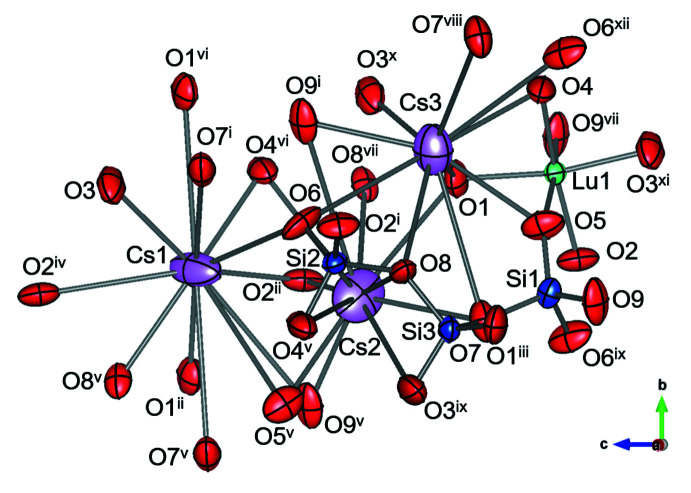
Atomic arrangement of Cs_3_LuSi_3_O_9_ shown with displacement ellipsoids at the 99% probability level. [Symmetry codes: (i) −*x* + 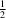 , *y* + 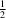 , *z* + 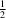 ; (ii) −*x*, −*y*, *z* + 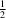 ; (iii) *x* + 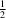 , −*y* + 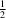 , *z*; (iv) *x*, *y*, *z* + 1; (v) −*x* + 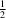 , *y* − 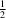 , *z* + 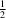 ; (vi) −*x*, 2 − *y*, *z* + 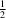 ; (vii) *x* − 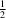 , −*y* + 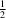 , *z*; (viii) *x*, *y* + 1, *z*; (ix) −*x* + 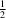 , *y* − 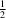 , *z* − 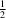 ; (x) −*x*, 1 − *y*, *z* − 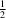 ; (xi) *x*, *y*, *z* − 1].

Cs_3_LuSi_3_O_9_ is a sechser single-chain silicate, which crystallizes in the ortho­rhom­bic space group, *Pna*2_1_. The chains of Si1-, Si2- and Si3-centered oxygen tetra­hedra are aligned along the [110] direction (Figs. 1 ▸ and 2 ▸). The period of the SiO_4_ zigzag chain is Si1–Si2–Si3–Si1–Si2–Si3, and the chain can be described as an unbranched **(** ***uB*** **)** single chain 1^1^_∞_ with a six-[SiO_4_]-tetra­hedra repeat unit [^6^Si_6_O_18_]: {uB,1^1^_∞_}[^6^Si_6_O_18_], in accordance with the classification of Liebau (1985 ▸). The Si—O_bridge_ bond lengths in the title compound lie in the range 1.650 (4)–1.664 (4) Å, and are slightly longer than the Si—O_terminal_ bond lengths [in the range 1.594 (4)–1.609 (4) Å; Table 1 ▸) and are in agreement with values found in other silicates. The bond-valence sums (BVSs) calculated for Si1, Si2, and Si3 using the bond-valence parameters presented by Gagné & Hawthorne (2015 ▸), are 3.903, 3.966, and 3.948, respectively, which closely match the value of 4 expected for Si^IV^.

The angles Si1—Si2—Si3, Si2—Si3—Si1 and Si3—Si1—Si2 in the SiO_4_-linked zigzag chain in Cs_3_LuSi_3_O_9_ are 133.26 (7), 128.41 (7) and 134.64 (7)°, respectively. These values are larger than the angles observed in K_6_Lu_2_Si_6_O_18_ (space group *Pm*2_1_ *n*, Filipenko *et al.*, 1988 ▸), a sechser single-chain silicate with an Si1–Si2–Si3–Si3–Si2–Si1 sequence (and angles: Si1—Si2—Si3 = 80.45, Si2—Si3—Si3 = 123.65 and Si3—Si3—Si2 = 123.65°), reflecting the difference in ionic size between Cs^+^ and K^+^.

**Figure 2:**
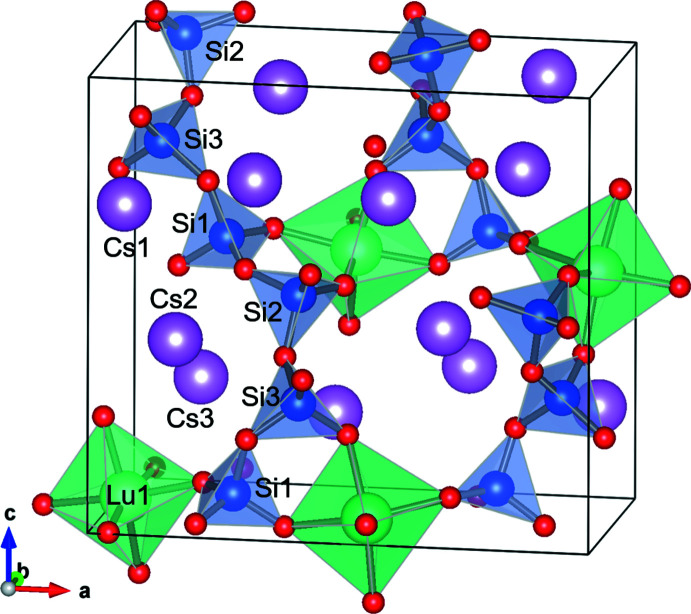
A polyhedral representation of Cs_3_LuSi_3_O_9_ showing the Lu1-centered oxygen octa­hedra (green) and Si-centered oxygen tetra­hedra (blue) linked together to form a 3-D framework with Cs ions (purple) contained in the framework voids.

Lu1 has sixfold coordination and is located in a distorted O octa­hedron, which connects two SiO_4_ single chains, as shown in Fig. 2 ▸. All the oxygen atoms of the Lu1-based octa­hedron are shared with SiO_4_ tetra­hedra. The Lu1—O bond distances range from 2.203 (4) to 2.255 (4) Å, and the average distance (2.2282 Å) is in good agreement with the corresponding value reported for K_6_Lu_2_Si_6_O_18_ (Filipenko *et al.*, 1988 ▸). A BVS value of 2.939 was obtained, which is close to the valence of 3 expected for Lu^III^.

The three distinct Cs sites, Cs1, Cs2 and Cs3, are situated in the voids of the three-dimensional framework composed of the silicate single chains connected by the Lu atoms. The Cs1 and Cs2 ions are surrounded by twelve O atoms, while the Cs3 ion is surrounded by ten O atoms. The BVS values for Cs1, Cs2, and Cs3 are 1.084, 0.919, 0.975, respectively [taking Cs—O distances in the range 2.869 (4)–4.228 (4) Å], which are close to the expected value of 1 for the Cs^I^ valence. All Cs sites are fully occupied, in contrast to the situation in the isotypic crystal structures of Cs_3_ *RE* ^III^Ge_3_O_9_ (Morrison *et al.*, 2019 ▸). When *RE* = Pr, Nd and Sm, disorder is observed in all three Cs sites and for the Eu–Yb analogues, disorder is observed in only two of the Cs sites.

The Madelung energy per formula unit for Cs_3_LuSi_3_O_9_, calculated using the structure parameters determined here within the *VESTA* program (Momma & Izumi, 2011 ▸), is −57,100 kJ mol^−1^, which is close to the value of −56,700 kJ mol^−1^ (difference *Δ* = 0.6%) calculated from the equation: 3/2Cs_2_O + 1/2 Lu_2_O_3_ + 3SiO_2_ = Cs_3_LuSi_3_O_9_ using enthalpy values within *VESTA* of −2,200, −15,600 and −15,200 kJ mol^−1^ for Cs_2_O, Lu_2_O_3_ and SiO_2_, respectively, together with crystal-structure data for Cs_2_O (Tsai *et al.*, 1956 ▸), Lu_2_O_3_ (Saiki *et al.*, 1985 ▸) and SiO_2_ (d’Amour *et al.*, 1979 ▸). The Madelung potentials for Cs1–Cs3 (−8.7 to −9.2 V), Lu1 (−32.8 V), and Si1–Si3 (−48.0 V) in Cs_3_LuSi_3_O_9_ are in good agreement with those obtained in *VESTA* for Cs (−9.0 V) in Cs_2_O, Lu (−31.3, −31.6 V) in Lu_2_O, and Si (−48.1 V) in SiO_2_.

## Synthesis and crystallization

Powders of Cs_2_CO_3_ (99.9%, Kojundo Chemical Lab. Co., Ltd.), Lu_2_O_3_ (99.999%, Nippon Yttrium Co., Ltd.) and SiO_2_ (99.999%, Mitsuwa Chemicals Co., Ltd.) were used as starting materials. The Lu_2_O_3_ and SiO_2_ powders were heated at 1273 K for 10 h in air, and stored in an air oven heated at 453 K, while the Cs_2_CO_3_ powder was heated at 673 K for 10 h and stored in an Ar-gas-filled glove box prior to use. 0.1955 g of Cs_2_CO_3_ powder were weighed in the glove box, and mixed with 0.0796 g of Lu_2_O_3_ and 0.00120 g of SiO_2_ (Cs:Lu:Si molar ratio = 3:1:0.5) in an agate mortar and pestle in air, and then pressed into a 5 mm diameter pellet. The pellet was heated at 1273 K for 3 h on a Pt plate in air, and cooled in the furnace by shutting off the power to the heater. The shape of the pellet was maintained; however, part of the sample had melted and solidified around the pellet on the Pt plate. Colourless transparent prismatic single crystals of around 0.1 mm in length were obtained from the melted part of the pellet. The unmelted part of the pellet consisted of Lu_2_O_3_ and Cs_3_LuSi_3_O_9_, which was verified by powder X-ray diffraction using a Bruker AXS, D2 PHASER diffractometer with Cu *K*α radiation.

## Refinement

Crystal data, data collection and structure refinement details are summarized in Table 2 ▸. Atomic coordinates and site labels were standardized with *Structure Tidy* (Gelato & Parthé, 1987 ▸) implemented in *PLATON* (Spek, 2020 ▸). The crystal structure was refined with consideration of twinning by inversion, which revealed a minor contribution of 3.1 (8)% for the inversion-related twin component.

## Supplementary Material

Crystal structure: contains datablock(s) global, I. DOI: 10.1107/S2056989021011439/cq2049sup1.cif

Structure factors: contains datablock(s) I. DOI: 10.1107/S2056989021011439/cq2049Isup2.hkl

CCDC reference: 2118472

Additional supporting information:  crystallographic information; 3D view; checkCIF report

## Figures and Tables

**Table 1:** Selected bond lengths (Å)

Cs1—O3	2.869 (4)	Cs3—O5	3.057 (4)
Cs1—O4^i^	2.972 (4)	Cs3—O4	3.066 (4)
Cs1—O1^ii^	3.028 (4)	Cs3—O3^viii^	3.335 (5)
Cs1—O8^iii^	3.069 (4)	Cs3—O8	3.354 (4)
Cs1—O2^ii^	3.074 (5)	Cs3—O6	3.599 (5)
Cs1—O7^iv^	3.485 (4)	Cs3—O6^ix^	3.857 (5)
Cs1—O2^v^	3.570 (5)	Cs3—O7^x^	3.942 (4)
Cs1—O9^iii^	4.044 (4)	Cs3—O7	4.030 (4)
Cs1—O1^i^	4.050 (4)	Lu1—O3^xi^	2.203 (4)
Cs1—O6	4.084 (4)	Lu1—O2	2.210 (4)
Cs1—O5^iii^	4.092 (4)	Lu1—O9^vi^	2.212 (4)
Cs1—O7^iii^	4.156 (4)	Lu1—O4	2.236 (4)
Cs1—O6^vi^	4.354 (4)	Lu1—O5	2.247 (4)
Cs2—O2^ii^	2.980 (4)	Lu1—O1	2.255 (4)
Cs2—O9^iii^	3.095 (4)	Si1—O5	1.606 (4)
Cs2—O6	3.130 (4)	Si1—O9	1.609 (4)
Cs2—O8	3.143 (4)	Si1—O7	1.661 (4)
Cs2—O5^iii^	3.170 (5)	Si1—O6^vii^	1.662 (4)
Cs2—O7	3.221 (4)	Si2—O4^iii^	1.599 (4)
Cs2—O3^vii^	3.752 (5)	Si2—O2^iv^	1.605 (4)
Cs2—O1	3.824 (4)	Si2—O6	1.650 (4)
Cs2—O4^iii^	3.926 (4)	Si2—O8	1.659 (4)
Cs2—O4^i^	4.106 (4)	Si3—O3^vii^	1.594 (4)
Cs2—O8^vi^	4.224 (4)	Si3—O1^xii^	1.608 (4)
Cs2—O9^iv^	4.228 (4)	Si3—O7	1.655 (4)
Cs3—O1	2.936 (4)	Si3—O8	1.664 (4)
Cs3—O9^iv^	2.983 (4)		

Symmetry codes: (i) -x, -y+1, z+{\script{1\over 2}}; (ii) -x, -y, z+{\script{1\over 2}}; (iii) -x+{\script{1\over 2}}, y-{\script{1\over 2}}, z+{\script{1\over 2}}; (iv) -x+{\script{1\over 2}}, y+{\script{1\over 2}}, z+{\script{1\over 2}}; (v) x, y, z+1; (vi) x-{\script{1\over 2}}, -y+{\script{1\over 2}}, z; (vii) -x+{\script{1\over 2}}, y-{\script{1\over 2}}, z-{\script{1\over 2}}; (viii) -x, -y+1, z-{\script{1\over 2}}; (ix) -x+{\script{1\over 2}}, y+{\script{1\over 2}}, z-{\script{1\over 2}}; (x) x, y+1, z; (xi) x, y, z-1; (xii) x+{\script{1\over 2}}, -y+{\script{1\over 2}}, z.

**Table 2:** Experimental details

Crystal data	
Chemical formula	Cs_3_LuSi_3_O_9_
*M*_r_	801.97
Crystal system, space group	Orthorhombic, *P* *n* *a*2_1_
Temperature (K)	300
*a*, *b*, *c* (Å)	13.3322 (3), 6.8618 (2), 12.2313 (3)
*V* (Å^3^)	1118.95 (5)
*Z*	4
Radiation type	Mo *K*α
μ (mm^−1^)	18.79
Crystal size (mm)	0.09 × 0.05 × 0.03

Data collection	
Diffractometer	Bruker APEXII CCD
Absorption correction	Multi-scan (*SADABS*; Bruker, 2018 ▸)
*T*_min_, *T*_max_	0.39, 0.58
No. of measured, independent and observed [*I* > 2σ(*I*)] reflections	24311, 3094, 3085
*R*_int_	0.034
(sin θ/λ)_max_ (Å^−1^)	0.695

Refinement	
*R*[*F* ^2^ > 2σ(*F* ^2^)], *wR*(*F* ^2^), *S*	0.014, 0.036, 1.11
No. of reflections	3094
No. of parameters	147
No. of restraints	1
Δρ_max_, Δρ_min_ (e Å^−3^)	1.87, −1.18
Absolute structure	Refined as an inversion twin
Absolute structure parameter	0.031 (8)

Computer programs: *BIS*, *APEX3* and *SAINT* (Bruker, 2018 ▸), *SHELXT2014/5* (Sheldrick, 2015*a* ▸), *SHELXL2014/7* (Sheldrick, 2015*b* ▸), *VESTA* (Momma & Izumi, 2011 ▸) and *publCIF* (Westrip, 2010 ▸).

## supplementary crystallographic information

### Crystal data

**Table:**

Cs_3_LuSi_3_O_9_	*D*_x_ = 4.761 Mg m^−^^3^
*M**_r_* = 801.97	Mo *K*α radiation, λ = 0.71073 Å
Orthorhombic, *P**n**a*2_1_	Cell parameters from 351 reflections
*a* = 13.3322 (3) Å	θ = 3.1–31.4°
*b* = 6.8618 (2) Å	µ = 18.79 mm^−^^1^
*c* = 12.2313 (3) Å	*T* = 300 K
*V* = 1118.95 (5) Å^3^	Prismatic, translucent colourless
*Z* = 4	0.09 × 0.05 × 0.03 mm
*F*(000) = 1400	

### Data collection

**Table:**

Bruker APEXII CCD diffractometer	3094 independent reflections
Radiation source: micro focus sealed tube	3085 reflections with *I* > 2σ(*I*)
Detector resolution: 7.3910 pixels mm^-1^	*R*_int_ = 0.034
φ and ω scans	θ_max_ = 29.6°, θ_min_ = 3.1°
Absorption correction: multi-scan (SADABS; Bruker, 2018)	*h* = −18→18
*T*_min_ = 0.39, *T*_max_ = 0.58	*k* = −9→9
24311 measured reflections	*l* = −16→16

### Refinement

**Table:**

Refinement on *F*^2^	*w* = 1/[σ^2^(*F*_o_^2^) + 2.7745*P*] where *P* = (*F*_o_^2^ + 2*F*_c_^2^)/3
Least-squares matrix: full	(Δ/σ)_max_ < 0.001
*R*[*F*^2^ > 2σ(*F*^2^)] = 0.014	Δρ_max_ = 1.87 e Å^−^^3^
*wR*(*F*^2^) = 0.036	Δρ_min_ = −1.18 e Å^−^^3^
*S* = 1.11	Extinction correction: SHELXL2017/1 (Sheldrick 2015b)
3094 reflections	Extinction coefficient: 0.00160 (14)
147 parameters	Absolute structure: Refined as an inversion twin
1 restraint	Absolute structure parameter: 0.031 (8)

### Special details

**Table:**

Geometry. All esds (except the esd in the dihedral angle between two l.s. planes) are estimated using the full covariance matrix. The cell esds are taken into account individually in the estimation of esds in distances, angles and torsion angles; correlations between esds in cell parameters are only used when they are defined by crystal symmetry. An approximate (isotropic) treatment of cell esds is used for estimating esds involving l.s. planes.
Refinement. Refined as a two-component inversion twin.

### Fractional atomic coordinates and isotropic or equivalent isotropic displacement parameters (Å^2^)

**Table:**

	*x*	*y*	*z*	*U*_iso_*/*U*_eq_	
Cs1	0.06219 (4)	0.10542 (6)	0.71151 (4)	0.02535 (10)	
Cs2	0.16894 (3)	0.05816 (7)	0.42547 (4)	0.02637 (11)	
Cs3	0.17391 (3)	0.55846 (6)	0.28251 (3)	0.02153 (9)	
Lu1	0.04370 (2)	0.34863 (3)	0.03915 (2)	0.00583 (6)	
Si1	0.28028 (11)	0.1098 (2)	0.08655 (12)	0.0070 (2)	
Si2	0.38805 (11)	0.3492 (2)	0.49763 (13)	0.0064 (2)	
Si3	0.41034 (10)	0.1056 (2)	0.29080 (12)	0.0068 (2)	
O1	0.0020 (3)	0.3178 (6)	0.2170 (3)	0.0118 (7)	
O2	0.0386 (3)	0.0346 (6)	−0.0005 (4)	0.0113 (7)	
O3	0.0643 (3)	0.4213 (6)	0.8652 (3)	0.0144 (8)	
O4	0.0722 (3)	0.6662 (6)	0.0659 (3)	0.0122 (8)	
O5	0.2041 (3)	0.2910 (6)	0.0854 (4)	0.0133 (8)	
O6	0.2752 (3)	0.4080 (5)	0.5419 (4)	0.0138 (7)	
O7	0.3124 (3)	0.0510 (6)	0.2136 (3)	0.0123 (7)	
O8	0.3669 (3)	0.2855 (6)	0.3688 (3)	0.0099 (7)	
O9	0.3794 (3)	0.1463 (6)	0.0142 (3)	0.0137 (9)	

### Atomic displacement parameters (Å^2^)

**Table:**

	*U*^11^	*U*^22^	*U*^33^	*U*^12^	*U*^13^	*U*^23^
Cs1	0.0370 (2)	0.01343 (17)	0.0257 (2)	0.00434 (16)	−0.01766 (18)	−0.00375 (16)
Cs2	0.02074 (19)	0.0252 (2)	0.0331 (2)	−0.00933 (15)	0.00863 (16)	−0.00168 (17)
Cs3	0.01763 (17)	0.0304 (2)	0.01654 (17)	0.00004 (14)	−0.00250 (13)	−0.00726 (16)
Lu1	0.00583 (9)	0.00579 (9)	0.00586 (9)	−0.00008 (6)	0.00064 (8)	0.00022 (8)
Si1	0.0049 (6)	0.0097 (6)	0.0063 (6)	−0.0004 (5)	−0.0006 (5)	−0.0016 (5)
Si2	0.0071 (6)	0.0052 (6)	0.0069 (5)	0.0001 (4)	0.0003 (5)	−0.0005 (5)
Si3	0.0076 (6)	0.0080 (6)	0.0047 (6)	0.0004 (5)	−0.0004 (5)	−0.0005 (5)
O1	0.0123 (17)	0.0161 (17)	0.0070 (16)	0.0002 (15)	0.0029 (15)	0.0011 (15)
O2	0.0087 (16)	0.0064 (16)	0.0189 (18)	0.0019 (13)	0.0002 (15)	−0.0011 (15)
O3	0.023 (2)	0.0113 (17)	0.0088 (18)	−0.0047 (16)	0.0032 (16)	0.0011 (15)
O4	0.020 (2)	0.0069 (17)	0.0092 (18)	0.0002 (14)	0.0028 (14)	0.0005 (13)
O5	0.0085 (17)	0.0122 (17)	0.019 (2)	0.0029 (14)	−0.0036 (14)	−0.0031 (16)
O6	0.0117 (16)	0.0112 (15)	0.0186 (18)	−0.0005 (13)	0.0073 (18)	−0.007 (2)
O7	0.0124 (17)	0.0167 (19)	0.0079 (17)	−0.0043 (14)	−0.0009 (14)	0.0010 (16)
O8	0.0121 (16)	0.0098 (16)	0.0077 (16)	0.0056 (14)	−0.0002 (14)	−0.0013 (14)
O9	0.0071 (16)	0.026 (2)	0.008 (2)	−0.0012 (15)	0.0023 (12)	0.0015 (14)

### Geometric parameters (Å, º)

**Table:**

Cs1—O3	2.869 (4)	Cs2—Si1^iv^	4.3209 (16)
Cs1—O4^i^	2.972 (4)	Cs2—Lu1^iii^	4.3218 (5)
Cs1—O1^ii^	3.028 (4)	Cs2—Si1	4.4174 (15)
Cs1—O8^iii^	3.069 (4)	Cs2—Si3^v^	4.4636 (15)
Cs1—O2^ii^	3.074 (5)	Cs3—O1	2.936 (4)
Cs1—O7^iv^	3.485 (4)	Cs3—O9^iv^	2.983 (4)
Cs1—Si2^v^	3.5115 (16)	Cs3—O5	3.057 (4)
Cs1—O2^vi^	3.570 (5)	Cs3—O4	3.066 (4)
Cs1—Si3^iii^	3.5829 (15)	Cs3—O3^ix^	3.335 (5)
Cs1—Si3^iv^	3.5852 (15)	Cs3—O8	3.354 (4)
Cs1—Cs3^iii^	3.6383 (6)	Cs3—O6	3.599 (5)
Cs1—Cs2	3.7909 (7)	Cs3—Si3^v^	3.6912 (14)
Cs1—Si2^iii^	3.9722 (16)	Cs3—Lu1	3.7345 (5)
Cs1—Cs3^i^	3.9977 (6)	Cs3—Si1^iv^	3.7851 (15)
Cs1—Lu1^ii^	4.0179 (5)	Cs3—O6^x^	3.857 (5)
Cs1—O9^iii^	4.044 (4)	Cs3—O7^xi^	3.942 (4)
Cs1—O1^i^	4.050 (4)	Cs3—O7	4.030 (4)
Cs1—O6	4.084 (4)	Cs3—Si2^x^	4.0992 (16)
Cs1—O5^iii^	4.092 (4)	Cs3—Si2	4.1396 (15)
Cs1—O7^iii^	4.156 (4)	Cs3—Si1	4.1512 (15)
Cs1—Cs2^ii^	4.1958 (7)	Cs3—Lu1^i^	4.3217 (5)
Cs1—Si1^iii^	4.2791 (15)	Cs3—O5^iv^	4.349 (4)
Cs1—Si1^iv^	4.3275 (16)	Cs3—O3^x^	4.405 (5)
Cs1—Lu1^vi^	4.3481 (5)	Cs3—Si3	4.4276 (15)
Cs1—O6^v^	4.354 (4)	Cs3—O2^iv^	4.665 (4)
Cs2—O2^ii^	2.980 (4)	Lu1—O3^xii^	2.203 (4)
Cs2—O9^iii^	3.095 (4)	Lu1—O2	2.210 (4)
Cs2—O6	3.130 (4)	Lu1—O9^v^	2.212 (4)
Cs2—O8	3.143 (4)	Lu1—O4	2.236 (4)
Cs2—O5^iii^	3.170 (5)	Lu1—O5	2.247 (4)
Cs2—O7	3.221 (4)	Lu1—O1	2.255 (4)
Cs2—Si3	3.6301 (15)	Si1—O5	1.606 (4)
Cs2—Si2	3.6470 (15)	Si1—O9	1.609 (4)
Cs2—Si1^iii^	3.7153 (16)	Si1—O7	1.661 (4)
Cs2—O3^vii^	3.752 (5)	Si1—O6^vii^	1.662 (4)
Cs2—O1	3.824 (4)	Si2—O4^iii^	1.599 (4)
Cs2—Cs3^viii^	3.8495 (6)	Si2—O2^iv^	1.605 (4)
Cs2—Cs3	3.8532 (7)	Si2—O6	1.650 (4)
Cs2—Si2^v^	3.8996 (15)	Si2—O8	1.659 (4)
Cs2—O4^iii^	3.926 (4)	Si3—O3^vii^	1.594 (4)
Cs2—O4^i^	4.106 (4)	Si3—O1^xiii^	1.608 (4)
Cs2—Lu1^ii^	4.2145 (5)	Si3—O7	1.655 (4)
Cs2—O8^v^	4.224 (4)	Si3—O8	1.664 (4)
Cs2—O9^iv^	4.228 (4)		
			
O3—Cs1—O4^i^	89.99 (12)	Cs2—Cs3—Lu1^i^	77.894 (10)
O3—Cs1—O1^ii^	135.45 (11)	O6^x^—Cs3—Lu1^i^	125.50 (6)
O4^i^—Cs1—O1^ii^	110.40 (11)	O7^xi^—Cs3—Lu1^i^	110.07 (6)
O3—Cs1—O8^iii^	97.28 (11)	Cs1^ix^—Cs3—Lu1^i^	62.878 (9)
O4^i^—Cs1—O8^iii^	159.80 (11)	O7—Cs3—Lu1^i^	125.93 (6)
O1^ii^—Cs1—O8^iii^	52.20 (10)	Si2^x^—Cs3—Lu1^i^	114.23 (2)
O3—Cs1—O2^ii^	142.44 (12)	Si2—Cs3—Lu1^i^	93.01 (2)
O4^i^—Cs1—O2^ii^	52.85 (11)	Si1—Cs3—Lu1^i^	139.30 (2)
O1^ii^—Cs1—O2^ii^	66.14 (11)	O1—Cs3—O5^iv^	136.94 (10)
O8^iii^—Cs1—O2^ii^	116.10 (11)	O9^iv^—Cs3—O5^iv^	37.42 (9)
O3—Cs1—O7^iv^	47.76 (10)	O5—Cs3—O5^iv^	147.45 (6)
O4^i^—Cs1—O7^iv^	80.27 (11)	O4—Cs3—O5^iv^	144.37 (9)
O1^ii^—Cs1—O7^iv^	167.63 (10)	O3^ix^—Cs3—O5^iv^	94.74 (9)
O8^iii^—Cs1—O7^iv^	118.38 (10)	O8—Cs3—O5^iv^	69.50 (9)
O2^ii^—Cs1—O7^iv^	119.34 (11)	O6—Cs3—O5^iv^	38.20 (8)
O3—Cs1—Si2^v^	115.30 (10)	Cs1^x^—Cs3—O5^iv^	79.00 (5)
O4^i^—Cs1—Si2^v^	26.94 (8)	Si3^v^—Cs3—O5^iv^	116.40 (6)
O1^ii^—Cs1—Si2^v^	85.11 (8)	Lu1—Cs3—O5^iv^	173.83 (5)
O8^iii^—Cs1—Si2^v^	137.25 (8)	Si1^iv^—Cs3—O5^iv^	21.35 (6)
O2^ii^—Cs1—Si2^v^	27.18 (8)	Cs2^xi^—Cs3—O5^iv^	44.99 (6)
O7^iv^—Cs1—Si2^v^	104.17 (7)	Cs2—Cs3—O5^iv^	86.95 (6)
O3—Cs1—O2^vi^	57.13 (11)	O6^x^—Cs3—O5^iv^	110.86 (8)
O4^i^—Cs1—O2^vi^	127.58 (10)	O7^xi^—Cs3—O5^iv^	72.09 (8)
O1^ii^—Cs1—O2^vi^	79.78 (10)	Cs1^ix^—Cs3—O5^iv^	105.54 (6)
O8^iii^—Cs1—O2^vi^	46.44 (10)	O7—Cs3—O5^iv^	108.90 (8)
O2^ii^—Cs1—O2^vi^	138.69 (4)	Si2^x^—Cs3—O5^iv^	128.38 (6)
O7^iv^—Cs1—O2^vi^	98.89 (10)	Si2—Cs3—O5^iv^	47.77 (6)
Si2^v^—Cs1—O2^vi^	133.55 (7)	Si1—Cs3—O5^iv^	129.50 (6)
O3—Cs1—Si3^iii^	123.02 (9)	Lu1^i^—Cs3—O5^iv^	65.06 (5)
O4^i^—Cs1—Si3^iii^	136.76 (8)	O1—Cs3—O3^x^	177.42 (10)
O1^ii^—Cs1—Si3^iii^	26.49 (8)	O9^iv^—Cs3—O3^x^	81.75 (10)
O8^iii^—Cs1—Si3^iii^	27.61 (8)	O5—Cs3—O3^x^	114.58 (10)
O2^ii^—Cs1—Si3^iii^	88.50 (8)	O4—Cs3—O3^x^	114.36 (10)
O7^iv^—Cs1—Si3^iii^	142.07 (7)	O3^ix^—Cs3—O3^x^	131.40 (8)
Si2^v^—Cs1—Si3^iii^	110.74 (3)	O8—Cs3—O3^x^	68.61 (9)
O2^vi^—Cs1—Si3^iii^	67.14 (7)	O6—Cs3—O3^x^	70.28 (8)
O3—Cs1—Si3^iv^	25.66 (9)	Cs1^x^—Cs3—O3^x^	40.39 (6)
O4^i^—Cs1—Si3^iv^	73.67 (8)	Si3^v^—Cs3—O3^x^	156.99 (6)
O1^ii^—Cs1—Si3^iv^	160.19 (8)	Lu1—Cs3—O3^x^	140.28 (6)
O8^iii^—Cs1—Si3^iv^	118.94 (8)	Si1^iv^—Cs3—O3^x^	66.05 (6)
O2^ii^—Cs1—Si3^iv^	124.88 (8)	Cs2^xi^—Cs3—O3^x^	53.56 (6)
O7^iv^—Cs1—Si3^iv^	27.02 (7)	Cs2—Cs3—O3^x^	114.39 (5)
Si2^v^—Cs1—Si3^iv^	100.61 (3)	O6^x^—Cs3—O3^x^	71.59 (8)
O2^vi^—Cs1—Si3^iv^	82.67 (7)	O7^xi^—Cs3—O3^x^	36.25 (8)
Si3^iii^—Cs1—Si3^iv^	146.38 (5)	Cs1^ix^—Cs3—O3^x^	110.35 (5)
O3—Cs1—Cs3^iii^	84.33 (9)	O7—Cs3—O3^x^	99.98 (8)
O4^i^—Cs1—Cs3^iii^	140.57 (9)	Si2^x^—Cs3—O3^x^	94.57 (6)
O1^ii^—Cs1—Cs3^iii^	100.56 (8)	Si2—Cs3—O3^x^	60.24 (6)
O8^iii^—Cs1—Cs3^iii^	59.28 (7)	Si1—Cs3—O3^x^	106.31 (6)
O2^ii^—Cs1—Cs3^iii^	126.61 (7)	Lu1^i^—Cs3—O3^x^	106.38 (6)
O7^iv^—Cs1—Cs3^iii^	67.16 (7)	O5^iv^—Cs3—O3^x^	45.64 (8)
Si2^v^—Cs1—Cs3^iii^	145.59 (3)	O1—Cs3—Si3	99.62 (8)
O2^vi^—Cs1—Cs3^iii^	80.65 (6)	O9^iv^—Cs3—Si3	106.83 (8)
Si3^iii^—Cs1—Cs3^iii^	75.63 (3)	O5—Cs3—Si3	60.20 (8)
Si3^iv^—Cs1—Cs3^iii^	85.49 (3)	O4—Cs3—Si3	120.26 (8)
O3—Cs1—Cs2	131.73 (8)	O3^ix^—Cs3—Si3	134.44 (8)
O4^i^—Cs1—Cs2	73.65 (8)	O8—Cs3—Si3	18.99 (7)
O1^ii^—Cs1—Cs2	92.56 (8)	O6—Cs3—Si3	60.75 (6)
O8^iii^—Cs1—Cs2	113.70 (7)	Cs1^x^—Cs3—Si3	51.62 (2)
O2^ii^—Cs1—Cs2	50.14 (8)	Si3^v^—Cs3—Si3	117.585 (15)
O7^iv^—Cs1—Cs2	84.35 (7)	Lu1—Cs3—Si3	94.51 (2)
Si2^v^—Cs1—Cs2	64.42 (3)	Si1^iv^—Cs3—Si3	85.87 (3)
O2^vi^—Cs1—Cs2	158.76 (6)	Cs2^xi^—Cs3—Si3	128.83 (2)
Si3^iii^—Cs1—Cs2	97.44 (3)	Cs2—Cs3—Si3	51.43 (2)
Si3^iv^—Cs1—Cs2	107.05 (3)	O6^x^—Cs3—Si3	109.18 (6)
Cs3^iii^—Cs1—Cs2	81.354 (14)	O7^xi^—Cs3—Si3	105.86 (6)
O3—Cs1—Si2^iii^	75.86 (9)	Cs1^ix^—Cs3—Si3	166.04 (2)
O4^i^—Cs1—Si2^iii^	149.52 (9)	O7—Cs3—Si3	21.92 (6)
O1^ii^—Cs1—Si2^iii^	66.62 (8)	Si2^x^—Cs3—Si3	120.30 (3)
O8^iii^—Cs1—Si2^iii^	22.99 (8)	Si2—Cs3—Si3	41.49 (3)
O2^ii^—Cs1—Si2^iii^	132.67 (8)	Si1—Cs3—Si3	41.37 (3)
O7^iv^—Cs1—Si2^iii^	107.56 (7)	Lu1^i^—Cs3—Si3	124.39 (2)
Si2^v^—Cs1—Si2^iii^	143.711 (19)	O5^iv^—Cs3—Si3	88.38 (6)
O2^vi^—Cs1—Si2^iii^	23.80 (7)	O3^x^—Cs3—Si3	80.05 (6)
Si3^iii^—Cs1—Si2^iii^	47.22 (3)	O1—Cs3—O2^iv^	140.96 (10)
Si3^iv^—Cs1—Si2^iii^	99.69 (3)	O9^iv^—Cs3—O2^iv^	70.26 (10)
Cs3^iii^—Cs1—Si2^iii^	65.73 (2)	O5—Cs3—O2^iv^	108.64 (9)
Cs2—Cs1—Si2^iii^	135.44 (3)	O4—Cs3—O2^iv^	149.71 (10)
O3—Cs1—Cs3^i^	55.21 (9)	O3^ix^—Cs3—O2^iv^	127.68 (9)
O4^i^—Cs1—Cs3^i^	49.57 (8)	O8—Cs3—O2^iv^	33.98 (8)
O1^ii^—Cs1—Cs3^i^	109.01 (8)	O6—Cs3—O2^iv^	34.93 (8)
O8^iii^—Cs1—Cs3^i^	121.27 (7)	Cs1^x^—Cs3—O2^iv^	49.04 (6)
O2^ii^—Cs1—Cs3^i^	91.10 (7)	Si3^v^—Cs3—O2^iv^	139.09 (6)
O7^iv^—Cs1—Cs3^i^	82.54 (7)	Lu1—Cs3—O2^iv^	145.27 (5)
Si2^v^—Cs1—Cs3^i^	65.80 (3)	Si1^iv^—Cs3—O2^iv^	46.50 (6)
O2^vi^—Cs1—Cs3^i^	78.16 (6)	Cs2^xi^—Cs3—O2^iv^	77.70 (5)
Si3^iii^—Cs1—Cs3^i^	125.02 (3)	Cs2—Cs3—O2^iv^	74.01 (5)
Si3^iv^—Cs1—Cs3^i^	57.95 (2)	O6^x^—Cs3—O2^iv^	108.15 (8)
Cs3^iii^—Cs1—Cs3^i^	139.522 (15)	O7^xi^—Cs3—O2^iv^	76.54 (8)
Cs2—Cs1—Cs3^i^	123.056 (15)	Cs1^ix^—Cs3—O2^iv^	142.26 (5)
Si2^iii^—Cs1—Cs3^i^	101.27 (2)	O7—Cs3—O2^iv^	73.28 (8)
O3—Cs1—Lu1^ii^	158.89 (9)	Si2^x^—Cs3—O2^iv^	131.79 (6)
O4^i^—Cs1—Lu1^ii^	83.27 (8)	Si2—Cs3—O2^iv^	19.87 (6)
O1^ii^—Cs1—Lu1^ii^	33.77 (8)	Si1—Cs3—O2^iv^	91.25 (6)
O8^iii^—Cs1—Lu1^ii^	83.25 (7)	Lu1^i^—Cs3—O2^iv^	98.25 (5)
O2^ii^—Cs1—Lu1^ii^	33.03 (8)	O5^iv^—Cs3—O2^iv^	38.83 (8)
O7^iv^—Cs1—Lu1^ii^	148.51 (7)	O3^x^—Cs3—O2^iv^	40.38 (7)
Si2^v^—Cs1—Lu1^ii^	56.33 (2)	Si3—Cs3—O2^iv^	51.49 (6)
O2^vi^—Cs1—Lu1^ii^	112.41 (7)	O3^xii^—Lu1—O2	90.72 (17)
Si3^iii^—Cs1—Lu1^ii^	55.64 (2)	O3^xii^—Lu1—O9^v^	89.23 (16)
Si3^iv^—Cs1—Lu1^ii^	156.94 (3)	O2—Lu1—O9^v^	87.41 (15)
Cs3^iii^—Cs1—Lu1^ii^	113.351 (13)	O3^xii^—Lu1—O4	84.26 (15)
Cs2—Cs1—Lu1^ii^	65.251 (10)	O2—Lu1—O4	171.01 (16)
Si2^iii^—Cs1—Lu1^ii^	100.24 (2)	O9^v^—Lu1—O4	99.95 (16)
Cs3^i^—Cs1—Lu1^ii^	106.540 (13)	O3^xii^—Lu1—O5	99.47 (17)
O3—Cs1—O9^iii^	167.84 (11)	O2—Lu1—O5	85.00 (15)
O4^i^—Cs1—O9^iii^	99.76 (10)	O9^v^—Lu1—O5	168.50 (16)
O1^ii^—Cs1—O9^iii^	47.21 (10)	O4—Lu1—O5	88.46 (16)
O8^iii^—Cs1—O9^iii^	75.96 (9)	O3^xii^—Lu1—O1	169.68 (16)
O2^ii^—Cs1—O9^iii^	48.52 (9)	O2—Lu1—O1	96.45 (16)
O7^iv^—Cs1—O9^iii^	126.54 (9)	O9^v^—Lu1—O1	83.71 (14)
Si2^v^—Cs1—O9^iii^	75.64 (6)	O4—Lu1—O1	89.54 (15)
O2^vi^—Cs1—O9^iii^	119.97 (9)	O5—Lu1—O1	88.57 (16)
Si3^iii^—Cs1—O9^iii^	52.85 (6)	O3^xii^—Lu1—Cs3	128.68 (11)
Si3^iv^—Cs1—O9^iii^	152.56 (7)	O2—Lu1—Cs3	124.41 (12)
Cs3^iii^—Cs1—O9^iii^	83.52 (6)	O9^v^—Lu1—Cs3	124.34 (10)
Cs2—Cs1—O9^iii^	46.40 (6)	O4—Lu1—Cs3	55.15 (11)
Si2^iii^—Cs1—O9^iii^	98.55 (6)	O5—Lu1—Cs3	54.89 (11)
Cs3^i^—Cs1—O9^iii^	136.95 (6)	O1—Lu1—Cs3	51.82 (10)
Lu1^ii^—Cs1—O9^iii^	31.85 (6)	O3^xii^—Lu1—Cs1^xiv^	136.55 (11)
O3—Cs1—O1^i^	41.66 (10)	O2—Lu1—Cs1^xiv^	49.32 (12)
O4^i^—Cs1—O1^i^	50.75 (9)	O9^v^—Lu1—Cs1^xiv^	74.72 (11)
O1^ii^—Cs1—O1^i^	151.28 (13)	O4—Lu1—Cs1^xiv^	137.59 (10)
O8^iii^—Cs1—O1^i^	138.93 (10)	O5—Lu1—Cs1^xiv^	93.78 (12)
O2^ii^—Cs1—O1^i^	103.12 (9)	O1—Lu1—Cs1^xiv^	48.28 (11)
O7^iv^—Cs1—O1^i^	40.89 (8)	Cs3—Lu1—Cs1^xiv^	92.527 (12)
Si2^v^—Cs1—O1^i^	77.64 (6)	O3^xii^—Lu1—Cs2^xiv^	85.11 (11)
O2^vi^—Cs1—O1^i^	95.63 (9)	O2—Lu1—Cs2^xiv^	42.36 (10)
Si3^iii^—Cs1—O1^i^	162.28 (6)	O9^v^—Lu1—Cs2^xiv^	45.48 (11)
Si3^iv^—Cs1—O1^i^	23.30 (6)	O4—Lu1—Cs2^xiv^	143.89 (12)
Cs3^iii^—Cs1—O1^i^	106.68 (6)	O5—Lu1—Cs2^xiv^	127.35 (11)
Cs2—Cs1—O1^i^	100.27 (6)	O1—Lu1—Cs2^xiv^	95.18 (11)
Si2^iii^—Cs1—O1^i^	116.95 (6)	Cs3—Lu1—Cs2^xiv^	146.201 (13)
Cs3^i^—Cs1—O1^i^	42.79 (6)	Cs1^xiv^—Lu1—Cs2^xiv^	54.773 (11)
Lu1^ii^—Cs1—O1^i^	133.81 (6)	O3^xii^—Lu1—Cs2^x^	60.24 (12)
O9^iii^—Cs1—O1^i^	144.29 (8)	O2—Lu1—Cs2^x^	106.33 (10)
O3—Cs1—O6	86.68 (10)	O9^v^—Lu1—Cs2^x^	145.93 (11)
O4^i^—Cs1—O6	81.18 (11)	O4—Lu1—Cs2^x^	64.69 (11)
O1^ii^—Cs1—O6	134.06 (9)	O5—Lu1—Cs2^x^	45.24 (12)
O8^iii^—Cs1—O6	117.91 (9)	O1—Lu1—Cs2^x^	124.05 (10)
O2^ii^—Cs1—O6	91.97 (10)	Cs3—Lu1—Cs2^x^	73.508 (9)
O7^iv^—Cs1—O6	39.11 (9)	Cs1^xiv^—Lu1—Cs2^x^	137.582 (10)
Si2^v^—Cs1—O6	92.07 (7)	Cs2^xiv^—Lu1—Cs2^x^	135.276 (14)
O2^vi^—Cs1—O6	129.18 (9)	O3^xii^—Lu1—Cs3^ix^	49.34 (12)
Si3^iii^—Cs1—O6	123.58 (6)	O2—Lu1—Cs3^ix^	87.93 (11)
Si3^iv^—Cs1—O6	65.15 (6)	O9^v^—Lu1—Cs3^ix^	39.89 (10)
Cs3^iii^—Cs1—O6	59.59 (6)	O4—Lu1—Cs3^ix^	94.39 (11)
Cs2—Cs1—O6	46.65 (6)	O5—Lu1—Cs3^ix^	147.96 (11)
Si2^iii^—Cs1—O6	123.81 (7)	O1—Lu1—Cs3^ix^	123.31 (10)
Cs3^i^—Cs1—O6	111.38 (5)	Cs3—Lu1—Cs3^ix^	146.522 (6)
Lu1^ii^—Cs1—O6	111.84 (6)	Cs1^xiv^—Lu1—Cs3^ix^	105.046 (10)
O9^iii^—Cs1—O6	87.65 (8)	Cs2^xiv^—Lu1—Cs3^ix^	53.592 (9)
O1^i^—Cs1—O6	70.04 (8)	Cs2^x^—Lu1—Cs3^ix^	108.201 (11)
O3—Cs1—O5^iii^	129.64 (11)	O3^xii^—Lu1—Cs1^xii^	35.84 (11)
O4^i^—Cs1—O5^iii^	120.73 (10)	O2—Lu1—Cs1^xii^	54.92 (12)
O1^ii^—Cs1—O5^iii^	73.63 (10)	O9^v^—Lu1—Cs1^xii^	86.28 (10)
O8^iii^—Cs1—O5^iii^	67.96 (10)	O4—Lu1—Cs1^xii^	119.97 (10)
O2^ii^—Cs1—O5^iii^	81.39 (9)	O5—Lu1—Cs1^xii^	96.34 (11)
O7^iv^—Cs1—O5^iii^	95.73 (9)	O1—Lu1—Cs1^xii^	150.11 (11)
Si2^v^—Cs1—O5^iii^	105.63 (7)	Cs3—Lu1—Cs1^xii^	148.898 (10)
O2^vi^—Cs1—O5^iii^	111.55 (9)	Cs1^xiv^—Lu1—Cs1^xii^	101.881 (6)
Si3^iii^—Cs1—O5^iii^	61.28 (7)	Cs2^xiv^—Lu1—Cs1^xii^	58.658 (10)
Si3^iv^—Cs1—O5^iii^	121.93 (6)	Cs2^x^—Lu1—Cs1^xii^	77.345 (10)
Cs3^iii^—Cs1—O5^iii^	46.12 (6)	Cs3^ix^—Lu1—Cs1^xii^	54.916 (9)
Cs2—Cs1—O5^iii^	47.24 (6)	O5—Si1—O9	113.2 (2)
Si2^iii^—Cs1—O5^iii^	88.37 (7)	O5—Si1—O7	111.1 (2)
Cs3^i^—Cs1—O5^iii^	170.29 (7)	O9—Si1—O7	109.9 (2)
Lu1^ii^—Cs1—O5^iii^	70.18 (6)	O5—Si1—O6^vii^	111.1 (2)
O9^iii^—Cs1—O5^iii^	38.52 (8)	O9—Si1—O6^vii^	108.3 (2)
O1^i^—Cs1—O5^iii^	133.04 (9)	O7—Si1—O6^vii^	102.7 (2)
O6—Cs1—O5^iii^	63.06 (8)	O5—Si1—Cs2^x^	57.96 (17)
O3—Cs1—O7^iii^	133.13 (11)	O9—Si1—Cs2^x^	55.27 (16)
O4^i^—Cs1—O7^iii^	136.79 (10)	O7—Si1—Cs2^x^	130.56 (15)
O1^ii^—Cs1—O7^iii^	40.17 (9)	O6^vii^—Si1—Cs2^x^	126.64 (19)
O8^iii^—Cs1—O7^iii^	38.49 (9)	O5—Si1—Cs3^vii^	99.52 (17)
O2^ii^—Cs1—O7^iii^	83.98 (10)	O9—Si1—Cs3^vii^	48.86 (15)
O7^iv^—Cs1—O7^iii^	127.58 (11)	O7—Si1—Cs3^vii^	148.73 (16)
Si2^v^—Cs1—O7^iii^	110.60 (6)	O6^vii^—Si1—Cs3^vii^	70.82 (18)
O2^vi^—Cs1—O7^iii^	84.53 (9)	Cs2^x^—Si1—Cs3^vii^	61.75 (3)
Si3^iii^—Cs1—O7^iii^	23.21 (6)	O5—Si1—Cs3	38.26 (16)
Si3^iv^—Cs1—O7^iii^	146.46 (6)	O9—Si1—Cs3	118.85 (17)
Cs3^iii^—Cs1—O7^iii^	61.85 (6)	O7—Si1—Cs3	74.22 (15)
Cs2—Cs1—O7^iii^	77.07 (6)	O6^vii^—Si1—Cs3	130.97 (16)
Si2^iii^—Cs1—O7^iii^	61.46 (6)	Cs2^x^—Si1—Cs3	75.78 (3)
Cs3^i^—Cs1—O7^iii^	147.53 (6)	Cs3^vii^—Si1—Cs3	133.75 (4)
Lu1^ii^—Cs1—O7^iii^	55.59 (6)	O5—Si1—Cs1^x^	72.45 (16)
O9^iii^—Cs1—O7^iii^	38.09 (8)	O9—Si1—Cs1^x^	70.73 (16)
O1^i^—Cs1—O7^iii^	168.40 (8)	O7—Si1—Cs1^x^	74.49 (15)
O6—Cs1—O7^iii^	100.87 (8)	O6^vii^—Si1—Cs1^x^	176.18 (16)
O5^iii^—Cs1—O7^iii^	38.11 (8)	Cs2^x^—Si1—Cs1^x^	56.08 (2)
O3—Cs1—Cs2^ii^	78.50 (8)	Cs3^vii^—Si1—Cs1^x^	110.23 (4)
O4^i^—Cs1—Cs2^ii^	94.13 (9)	Cs3—Si1—Cs1^x^	51.107 (19)
O1^ii^—Cs1—Cs2^ii^	61.44 (8)	O5—Si1—Cs2^vii^	140.66 (16)
O8^iii^—Cs1—Cs2^ii^	69.12 (7)	O9—Si1—Cs2^vii^	75.93 (16)
O2^ii^—Cs1—Cs2^ii^	96.98 (8)	O7—Si1—Cs2^vii^	99.98 (15)
O7^iv^—Cs1—Cs2^ii^	125.63 (7)	O6^vii^—Si1—Cs2^vii^	35.89 (14)
Si2^v^—Cs1—Cs2^ii^	90.16 (3)	Cs2^x^—Si1—Cs2^vii^	117.07 (4)
O2^vi^—Cs1—Cs2^ii^	44.23 (6)	Cs3^vii^—Si1—Cs2^vii^	56.30 (2)
Si3^iii^—Cs1—Cs2^ii^	69.52 (3)	Cs3—Si1—Cs2^vii^	165.12 (4)
Si3^iv^—Cs1—Cs2^ii^	99.35 (3)	Cs1^x^—Si1—Cs2^vii^	141.46 (4)
Cs3^iii^—Cs1—Cs2^ii^	122.524 (16)	O5—Si1—Cs1^vii^	158.36 (17)
Cs2—Cs1—Cs2^ii^	145.944 (14)	O9—Si1—Cs1^vii^	85.41 (16)
Si2^iii^—Cs1—Cs2^ii^	56.96 (2)	O7—Si1—Cs1^vii^	49.49 (15)
Cs3^i^—Cs1—Cs2^ii^	55.983 (11)	O6^vii^—Si1—Cs1^vii^	70.45 (15)
Lu1^ii^—Cs1—Cs2^ii^	82.056 (12)	Cs2^x^—Si1—Cs1^vii^	139.56 (4)
O9^iii^—Cs1—Cs2^ii^	107.77 (6)	Cs3^vii^—Si1—Cs1^vii^	101.20 (3)
O1^i^—Cs1—Cs2^ii^	95.50 (6)	Cs3—Si1—Cs1^vii^	123.71 (4)
O6—Cs1—Cs2^ii^	164.48 (6)	Cs1^x^—Si1—Cs1^vii^	105.74 (3)
O5^iii^—Cs1—Cs2^ii^	130.80 (6)	Cs2^vii^—Si1—Cs1^vii^	51.995 (19)
O7^iii^—Cs1—Cs2^ii^	92.69 (6)	O5—Si1—Cs2	81.83 (17)
O3—Cs1—Si1^iii^	146.06 (10)	O9—Si1—Cs2	143.52 (16)
O4^i^—Cs1—Si1^iii^	120.06 (8)	O7—Si1—Cs2	35.83 (15)
O1^ii^—Cs1—Si1^iii^	52.03 (8)	O6^vii^—Si1—Cs2	95.27 (19)
O8^iii^—Cs1—Si1^iii^	60.28 (8)	Cs2^x^—Si1—Cs2	128.71 (4)
O2^ii^—Cs1—Si1^iii^	70.43 (8)	Cs3^vii^—Si1—Cs2	165.63 (4)
O7^iv^—Cs1—Si1^iii^	117.66 (7)	Cs3—Si1—Cs2	53.34 (2)
Si2^v^—Cs1—Si1^iii^	97.41 (3)	Cs1^x^—Si1—Cs2	83.89 (3)
O2^vi^—Cs1—Si1^iii^	106.70 (7)	Cs2^vii^—Si1—Cs2	114.22 (3)
Si3^iii^—Cs1—Si1^iii^	44.42 (3)	Cs1^vii^—Si1—Cs2	76.55 (3)
Si3^iv^—Cs1—Si1^iii^	143.83 (3)	O5—Si1—Cs3^viii^	115.92 (17)
Cs3^iii^—Cs1—Si1^iii^	62.63 (2)	O9—Si1—Cs3^viii^	130.79 (17)
Cs2—Cs1—Si1^iii^	54.42 (2)	O7—Si1—Cs3^viii^	53.48 (14)
Si2^iii^—Cs1—Si1^iii^	83.16 (3)	O6^vii^—Si1—Cs3^viii^	50.38 (18)
Cs3^i^—Cs1—Si1^iii^	157.30 (2)	Cs2^x^—Si1—Cs3^viii^	172.92 (4)
Lu1^ii^—Cs1—Si1^iii^	50.86 (2)	Cs3^vii^—Si1—Cs3^viii^	118.36 (4)
O9^iii^—Cs1—Si1^iii^	22.07 (6)	Cs3—Si1—Cs3^viii^	101.51 (3)
O1^i^—Cs1—Si1^iii^	152.41 (6)	Cs1^x^—Si1—Cs3^viii^	127.30 (3)
O6—Cs1—Si1^iii^	83.18 (6)	Cs2^vii^—Si1—Cs3^viii^	64.81 (2)
O5^iii^—Cs1—Si1^iii^	21.97 (6)	Cs1^vii^—Si1—Cs3^viii^	47.326 (17)
O7^iii^—Cs1—Si1^iii^	22.66 (6)	Cs2—Si1—Cs3^viii^	49.844 (18)
Cs2^ii^—Cs1—Si1^iii^	111.76 (2)	O4^iii^—Si2—O2^iv^	114.4 (2)
O3—Cs1—Si1^iv^	67.82 (8)	O4^iii^—Si2—O6	108.9 (2)
O4^i^—Cs1—Si1^iv^	70.07 (9)	O2^iv^—Si2—O6	110.9 (2)
O1^ii^—Cs1—Si1^iv^	155.79 (8)	O4^iii^—Si2—O8	110.2 (2)
O8^iii^—Cs1—Si1^iv^	130.11 (7)	O2^iv^—Si2—O8	109.0 (2)
O2^ii^—Cs1—Si1^iv^	99.45 (8)	O6—Si2—O8	102.8 (2)
O7^iv^—Cs1—Si1^iv^	21.25 (7)	O4^iii^—Si2—Cs1^xiii^	57.38 (15)
Si2^v^—Cs1—Si1^iv^	89.24 (3)	O2^iv^—Si2—Cs1^xiii^	61.05 (17)
O2^vi^—Cs1—Si1^iv^	120.01 (7)	O6—Si2—Cs1^xiii^	109.69 (19)
Si3^iii^—Cs1—Si1^iv^	144.25 (3)	O8—Si2—Cs1^xiii^	147.47 (15)
Si3^iv^—Cs1—Si1^iv^	43.97 (3)	O4^iii^—Si2—Cs2	87.84 (16)
Cs3^iii^—Cs1—Si1^iv^	71.69 (2)	O2^iv^—Si2—Cs2	157.77 (17)
Cs2—Cs1—Si1^iv^	63.91 (2)	O6—Si2—Cs2	58.84 (15)
Si2^iii^—Cs1—Si1^iv^	125.74 (3)	O8—Si2—Cs2	59.33 (14)
Cs3^i^—Cs1—Si1^iv^	89.85 (2)	Cs1^xiii^—Si2—Cs2	139.33 (5)
Lu1^ii^—Cs1—Si1^iv^	127.26 (2)	O4^iii^—Si2—Cs2^xiii^	85.86 (17)
O9^iii^—Cs1—Si1^iv^	108.59 (6)	O2^iv^—Si2—Cs2^xiii^	44.67 (15)
O1^i^—Cs1—Si1^iv^	47.69 (6)	O6—Si2—Cs2^xiii^	155.59 (15)
O6—Cs1—Si1^iv^	22.56 (6)	O8—Si2—Cs2^xiii^	89.49 (14)
O5^iii^—Cs1—Si1^iv^	85.35 (6)	Cs1^xiii^—Si2—Cs2^xiii^	61.26 (3)
O7^iii^—Cs1—Si1^iv^	122.62 (6)	Cs2—Si2—Cs2^xiii^	143.49 (4)
Cs2^ii^—Cs1—Si1^iv^	142.25 (2)	O4^iii^—Si2—Cs1^x^	138.82 (17)
Si1^iii^—Cs1—Si1^iv^	105.74 (3)	O2^iv^—Si2—Cs1^x^	63.90 (17)
O3—Cs1—Lu1^vi^	26.72 (8)	O6—Si2—Cs1^x^	109.48 (18)
O4^i^—Cs1—Lu1^vi^	108.40 (8)	O8—Si2—Cs1^x^	46.25 (14)
O1^ii^—Cs1—Lu1^vi^	109.36 (8)	Cs1^xiii^—Si2—Cs1^x^	120.44 (4)
O8^iii^—Cs1—Lu1^vi^	73.39 (7)	Cs2—Si2—Cs1^x^	99.38 (4)
O2^ii^—Cs1—Lu1^vi^	150.75 (7)	Cs2^xiii^—Si2—Cs1^x^	64.41 (3)
O7^iv^—Cs1—Lu1^vi^	71.56 (7)	O4^iii^—Si2—Cs3^iii^	40.57 (16)
Si2^v^—Cs1—Lu1^vi^	127.96 (3)	O2^iv^—Si2—Cs3^iii^	119.76 (18)
O2^vi^—Cs1—Lu1^vi^	30.44 (7)	O6—Si2—Cs3^iii^	69.88 (18)
Si3^iii^—Cs1—Lu1^vi^	97.11 (3)	O8—Si2—Cs3^iii^	130.14 (15)
Si3^iv^—Cs1—Lu1^vi^	52.34 (2)	Cs1^xiii^—Si2—Cs3^iii^	62.82 (3)
Cs3^iii^—Cs1—Lu1^vi^	82.461 (11)	Cs2—Si2—Cs3^iii^	77.16 (3)
Cs2—Cs1—Lu1^vi^	154.785 (14)	Cs2^xiii^—Si2—Cs3^iii^	117.73 (4)
Si2^iii^—Cs1—Lu1^vi^	50.75 (2)	Cs1^x^—Si2—Cs3^iii^	176.34 (4)
Cs3^i^—Cs1—Lu1^vi^	62.206 (10)	O4^iii^—Si2—Cs3	146.46 (17)
Lu1^ii^—Cs1—Lu1^vi^	139.577 (14)	O2^iv^—Si2—Cs3	98.88 (17)
O9^iii^—Cs1—Lu1^vi^	149.30 (6)	O6—Si2—Cs3	59.64 (19)
O1^i^—Cs1—Lu1^vi^	66.27 (6)	O8—Si2—Cs3	51.01 (13)
O6—Cs1—Lu1^vi^	108.21 (6)	Cs1^xiii^—Si2—Cs3	153.99 (4)
O5^iii^—Cs1—Lu1^vi^	126.37 (6)	Cs2—Si2—Cs3	58.92 (2)
O7^iii^—Cs1—Lu1^vi^	111.62 (6)	Cs2^xiii^—Si2—Cs3	117.51 (4)
Cs2^ii^—Cs1—Lu1^vi^	59.080 (10)	Cs1^x^—Si2—Cs3	53.25 (2)
Si1^iii^—Cs1—Lu1^vi^	131.45 (2)	Cs3^iii^—Si2—Cs3	124.76 (4)
Si1^iv^—Cs1—Lu1^vi^	92.64 (2)	O4^iii^—Si2—Cs1	76.58 (17)
O3—Cs1—O6^v^	109.67 (11)	O2^iv^—Si2—Cs1	137.47 (17)
O4^i^—Cs1—O6^v^	36.49 (10)	O6—Si2—Cs1	34.26 (15)
O1^ii^—Cs1—O6^v^	75.04 (9)	O8—Si2—Cs1	104.15 (15)
O8^iii^—Cs1—O6^v^	123.67 (9)	Cs1^xiii^—Si2—Cs1	101.58 (3)
O2^ii^—Cs1—O6^v^	37.56 (9)	Cs2—Si2—Cs1	45.200 (19)
O7^iv^—Cs1—O6^v^	116.34 (8)	Cs2^xiii^—Si2—Cs1	160.60 (4)
Si2^v^—Cs1—O6^v^	20.90 (6)	Cs1^x^—Si2—Cs1	134.92 (4)
O2^vi^—Cs1—O6^v^	112.95 (9)	Cs3^iii^—Si2—Cs1	42.877 (16)
Si3^iii^—Cs1—O6^v^	101.46 (5)	Cs3—Si2—Cs1	81.89 (2)
Si3^iv^—Cs1—O6^v^	103.82 (6)	O4^iii^—Si2—Cs3^xiii^	67.32 (16)
Cs3^iii^—Cs1—O6^v^	164.12 (6)	O2^iv^—Si2—Cs3^xiii^	89.29 (16)
Cs2—Cs1—O6^v^	83.59 (6)	O6—Si2—Cs3^xiii^	158.25 (15)
Si2^iii^—Cs1—O6^v^	123.87 (6)	O8—Si2—Cs3^xiii^	61.41 (14)
Cs3^i^—Cs1—O6^v^	54.80 (6)	Cs1^xiii^—Si2—Cs3^xiii^	86.70 (3)
Lu1^ii^—Cs1—O6^v^	54.89 (5)	Cs2—Si2—Cs3^xiii^	99.41 (3)
O9^iii^—Cs1—O6^v^	82.45 (8)	Cs2^xiii^—Si2—Cs3^xiii^	45.395 (17)
O1^i^—Cs1—O6^v^	80.95 (7)	Cs1^x^—Si2—Cs3^xiii^	71.50 (2)
O6—Cs1—O6^v^	112.32 (11)	Cs3^iii^—Si2—Cs3^xiii^	107.68 (3)
O5^iii^—Cs1—O6^v^	118.59 (9)	Cs3—Si2—Cs3^xiii^	110.86 (3)
O7^iii^—Cs1—O6^v^	109.72 (8)	Cs1—Si2—Cs3^xiii^	130.50 (3)
Cs2^ii^—Cs1—O6^v^	69.27 (6)	O3^vii^—Si3—O1^xiii^	114.7 (2)
Si1^iii^—Cs1—O6^v^	104.15 (6)	O3^vii^—Si3—O7	108.3 (2)
Si1^iv^—Cs1—O6^v^	105.97 (6)	O1^xiii^—Si3—O7	110.7 (2)
Lu1^vi^—Cs1—O6^v^	113.42 (6)	O3^vii^—Si3—O8	109.6 (2)
O2^ii^—Cs2—O9^iii^	60.35 (11)	O1^xiii^—Si3—O8	110.1 (2)
O2^ii^—Cs2—O6	116.46 (11)	O7—Si3—O8	102.7 (2)
O9^iii^—Cs2—O6	129.40 (12)	O3^vii^—Si3—Cs1^x^	153.15 (18)
O2^ii^—Cs2—O8	162.29 (11)	O1^xiii^—Si3—Cs1^x^	57.13 (16)
O9^iii^—Cs2—O8	134.84 (10)	O7—Si3—Cs1^x^	98.21 (15)
O6—Cs2—O8	48.68 (10)	O8—Si3—Cs1^x^	58.73 (14)
O2^ii^—Cs2—O5^iii^	100.65 (11)	O3^vii^—Si3—Cs1^vii^	51.18 (16)
O9^iii^—Cs2—O5^iii^	50.71 (10)	O1^xiii^—Si3—Cs1^vii^	94.80 (16)
O6—Cs2—O5^iii^	85.47 (11)	O7—Si3—Cs1^vii^	73.12 (15)
O8—Cs2—O5^iii^	88.54 (10)	O8—Si3—Cs1^vii^	154.24 (16)
O2^ii^—Cs2—O7	142.42 (12)	Cs1^x^—Si3—Cs1^vii^	146.38 (5)
O9^iii^—Cs2—O7	113.07 (10)	O3^vii^—Si3—Cs2	81.85 (18)
O6—Cs2—O7	96.27 (10)	O1^xiii^—Si3—Cs2	163.35 (17)
O8—Cs2—O7	48.07 (10)	O7—Si3—Cs2	62.53 (15)
O5^iii^—Cs2—O7	99.82 (11)	O8—Si3—Cs2	59.86 (14)
O2^ii^—Cs2—Si3	168.59 (9)	Cs1^x^—Si3—Cs2	107.41 (4)
O9^iii^—Cs2—Si3	115.18 (8)	Cs1^vii^—Si3—Cs2	97.32 (4)
O6—Cs2—Si3	74.72 (8)	O3^vii^—Si3—Cs3^xiii^	64.63 (17)
O8—Cs2—Si3	27.25 (7)	O1^xiii^—Si3—Cs3^xiii^	50.25 (15)
O5^iii^—Cs2—Si3	81.86 (8)	O7—Si3—Cs3^xiii^	131.83 (16)
O7—Cs2—Si3	27.12 (7)	O8—Si3—Cs3^xiii^	124.96 (15)
O2^ii^—Cs2—Si2	141.91 (9)	Cs1^x^—Si3—Cs3^xiii^	100.79 (4)
O9^iii^—Cs2—Si2	125.57 (8)	Cs1^vii^—Si3—Cs3^xiii^	66.63 (3)
O6—Cs2—Si2	26.81 (7)	Cs2—Si3—Cs3^xiii^	146.01 (5)
O8—Cs2—Si2	27.00 (7)	O3^vii^—Si3—Cs3	136.15 (18)
O5^iii^—Cs2—Si2	74.91 (8)	O1^xiii^—Si3—Cs3	107.39 (16)
O7—Cs2—Si2	74.19 (7)	O7—Si3—Cs3	65.40 (15)
Si3—Cs2—Si2	49.50 (3)	O8—Si3—Cs3	40.97 (13)
O2^ii^—Cs2—Si1^iii^	80.29 (8)	Cs1^x^—Si3—Cs3	52.75 (2)
O9^iii^—Cs2—Si1^iii^	25.30 (8)	Cs1^vii^—Si3—Cs3	137.60 (4)
O6—Cs2—Si1^iii^	108.13 (9)	Cs2—Si3—Cs3	56.09 (2)
O8—Cs2—Si1^iii^	112.01 (7)	Cs3^xiii^—Si3—Cs3	153.02 (4)
O5^iii^—Cs2—Si1^iii^	25.42 (8)	O3^vii^—Si3—Cs2^xiii^	92.04 (17)
O7—Cs2—Si1^iii^	107.81 (8)	O1^xiii^—Si3—Cs2^xiii^	56.69 (15)
Si3—Cs2—Si1^iii^	98.82 (3)	O7—Si3—Cs2^xiii^	159.60 (15)
Si2—Cs2—Si1^iii^	100.32 (3)	O8—Si3—Cs2^xiii^	70.95 (14)
O2^ii^—Cs2—O3^vii^	152.44 (10)	Cs1^x^—Si3—Cs2^xiii^	61.71 (2)
O9^iii^—Cs2—O3^vii^	92.16 (10)	Cs1^vii^—Si3—Cs2^xiii^	121.05 (4)
O6—Cs2—O3^vii^	81.52 (9)	Cs2—Si3—Cs2^xiii^	124.31 (4)
O8—Cs2—O3^vii^	44.34 (10)	Cs3^xiii^—Si3—Cs2^xiii^	55.42 (2)
O5^iii^—Cs2—O3^vii^	58.02 (10)	Cs3—Si3—Cs2^xiii^	101.28 (3)
O7—Cs2—O3^vii^	43.55 (10)	O3^vii^—Si3—Cs3^viii^	62.66 (17)
Si3—Cs2—O3^vii^	24.87 (7)	O1^xiii^—Si3—Cs3^viii^	136.69 (16)
Si2—Cs2—O3^vii^	54.92 (7)	O7—Si3—Cs3^viii^	46.17 (14)
Si1^iii^—Cs2—O3^vii^	73.99 (7)	O8—Si3—Cs3^viii^	110.86 (15)
O2^ii^—Cs2—Cs1	52.35 (9)	Cs1^x^—Si3—Cs3^viii^	142.47 (4)
O9^iii^—Cs2—Cs1	71.11 (8)	Cs1^vii^—Si3—Cs3^viii^	47.709 (19)
O6—Cs2—Cs1	71.60 (8)	Cs2—Si3—Cs3^viii^	51.00 (2)
O8—Cs2—Cs1	118.44 (7)	Cs3^xiii^—Si3—Cs3^viii^	112.21 (3)
O5^iii^—Cs2—Cs1	71.37 (8)	Cs3—Si3—Cs3^viii^	94.54 (3)
O7—Cs2—Cs1	165.18 (7)	Cs2^xiii^—Si3—Cs3^viii^	154.16 (4)
Si3—Cs2—Cs1	138.07 (3)	O3^vii^—Si3—Cs2^x^	160.88 (17)
Si2—Cs2—Cs1	91.75 (3)	O1^xiii^—Si3—Cs2^x^	60.62 (16)
Si1^iii^—Cs2—Cs1	69.50 (2)	O7—Si3—Cs2^x^	61.07 (14)
O3^vii^—Cs2—Cs1	123.97 (7)	O8—Si3—Cs2^x^	88.89 (14)
O2^ii^—Cs2—O1	76.22 (10)	Cs1^x^—Si3—Cs2^x^	42.697 (19)
O9^iii^—Cs2—O1	122.55 (10)	Cs1^vii^—Si3—Cs2^x^	109.74 (3)
O6—Cs2—O1	102.09 (11)	Cs2—Si3—Cs2^x^	104.30 (3)
O8—Cs2—O1	96.35 (9)	Cs3^xiii^—Si3—Cs2^x^	109.29 (3)
O5^iii^—Cs2—O1	172.44 (10)	Cs3—Si3—Cs2^x^	56.810 (18)
O7—Cs2—O1	79.40 (9)	Cs2^xiii^—Si3—Cs2^x^	98.93 (3)
Si3—Cs2—O1	99.88 (6)	Cs3^viii^—Si3—Cs2^x^	106.84 (3)
Si2—Cs2—O1	111.86 (7)	Si3^v^—O1—Lu1	134.3 (2)
Si1^iii^—Cs2—O1	147.70 (7)	Si3^v^—O1—Cs3	104.8 (2)
O3^vii^—Cs2—O1	122.49 (9)	Lu1—O1—Cs3	91.04 (13)
Cs1—Cs2—O1	110.92 (6)	Si3^v^—O1—Cs1^xiv^	96.38 (18)
O2^ii^—Cs2—Cs3^viii^	87.93 (8)	Lu1—O1—Cs1^xiv^	97.95 (14)
O9^iii^—Cs2—Cs3^viii^	49.43 (7)	Cs3—O1—Cs1^xiv^	140.01 (15)
O6—Cs2—Cs3^viii^	151.89 (7)	Si3^v^—O1—Cs2	102.74 (17)
O8—Cs2—Cs3^viii^	109.11 (7)	Lu1—O1—Cs2	122.93 (14)
O5^iii^—Cs2—Cs3^viii^	75.88 (8)	Cs3—O1—Cs2	68.04 (8)
O7—Cs2—Cs3^viii^	67.09 (7)	Cs1^xiv^—O1—Cs2	74.50 (9)
Si3—Cs2—Cs3^viii^	81.86 (3)	Si3^v^—O1—Cs1^ix^	61.90 (14)
Si2—Cs2—Cs3^viii^	125.72 (3)	Lu1—O1—Cs1^ix^	86.80 (12)
Si1^iii^—Cs2—Cs3^viii^	60.02 (2)	Cs3—O1—Cs1^ix^	67.65 (8)
O3^vii^—Cs2—Cs3^viii^	70.81 (7)	Cs1^xiv^—O1—Cs1^ix^	151.28 (13)
Cs1—Cs2—Cs3^viii^	120.123 (14)	Cs2—O1—Cs1^ix^	126.11 (11)
O1—Cs2—Cs3^viii^	97.01 (7)	Si3^v^—O1—Cs2^xiv^	102.96 (17)
O2^ii^—Cs2—Cs3	110.12 (8)	Lu1—O1—Cs2^xiv^	57.87 (9)
O9^iii^—Cs2—Cs3	167.48 (8)	Cs3—O1—Cs2^xiv^	148.00 (12)
O6—Cs2—Cs3	61.02 (9)	Cs1^xiv^—O1—Cs2^xiv^	49.83 (6)
O8—Cs2—Cs3	56.18 (7)	Cs2—O1—Cs2^xiv^	120.32 (10)
O5^iii^—Cs2—Cs3	141.80 (8)	Cs1^ix^—O1—Cs2^xiv^	113.54 (9)
O7—Cs2—Cs3	68.79 (7)	Si3^v^—O1—Cs3^v^	55.25 (14)
Si3—Cs2—Cs3	72.48 (2)	Lu1—O1—Cs3^v^	114.01 (13)
Si2—Cs2—Cs3	66.93 (2)	Cs3—O1—Cs3^v^	154.67 (12)
Si1^iii^—Cs2—Cs3	167.22 (3)	Cs1^xiv^—O1—Cs3^v^	44.07 (5)
O3^vii^—Cs2—Cs3	96.75 (7)	Cs2—O1—Cs3^v^	99.05 (8)
Cs1—Cs2—Cs3	110.429 (14)	Cs1^ix^—O1—Cs3^v^	108.26 (8)
O1—Cs2—Cs3	44.97 (6)	Cs2^xiv^—O1—Cs3^v^	57.29 (5)
Cs3^viii^—Cs2—Cs3	125.956 (16)	Si3^v^—O1—Cs2^v^	11.56 (12)
O2^ii^—Cs2—Si2^v^	22.24 (8)	Lu1—O1—Cs2^v^	132.00 (14)
O9^iii^—Cs2—Si2^v^	82.51 (8)	Cs3—O1—Cs2^v^	116.19 (11)
O6—Cs2—Si2^v^	101.92 (7)	Cs1^xiv^—O1—Cs2^v^	85.77 (8)
O8—Cs2—Si2^v^	140.84 (7)	Cs2—O1—Cs2^v^	104.28 (8)
O5^iii^—Cs2—Si2^v^	117.87 (8)	Cs1^ix^—O1—Cs2^v^	70.55 (6)
O7—Cs2—Si2^v^	138.99 (8)	Cs2^xiv^—O1—Cs2^v^	92.60 (7)
Si3—Cs2—Si2^v^	159.96 (3)	Cs3^v^—O1—Cs2^v^	43.78 (3)
Si2—Cs2—Si2^v^	128.69 (3)	Si2^vii^—O2—Lu1	139.2 (2)
Si1^iii^—Cs2—Si2^v^	100.95 (3)	Si2^vii^—O2—Cs2^xiv^	113.1 (2)
O3^vii^—Cs2—Si2^v^	174.67 (7)	Lu1—O2—Cs2^xiv^	107.66 (14)
Cs1—Cs2—Si2^v^	54.31 (2)	Si2^vii^—O2—Cs1^xiv^	91.77 (19)
O1—Cs2—Si2^v^	61.06 (6)	Lu1—O2—Cs1^xiv^	97.65 (15)
Cs3^viii^—Cs2—Si2^v^	105.34 (2)	Cs2^xiv^—O2—Cs1^xiv^	77.51 (10)
Cs3—Cs2—Si2^v^	88.51 (2)	Si2^vii^—O2—Cs1^xii^	92.29 (19)
O2^ii^—Cs2—O4^iii^	136.35 (11)	Lu1—O2—Cs1^xii^	94.64 (14)
O9^iii^—Cs2—O4^iii^	101.65 (10)	Cs2^xiv^—O2—Cs1^xii^	79.10 (10)
O6—Cs2—O4^iii^	42.12 (9)	Cs1^xiv^—O2—Cs1^xii^	155.96 (14)
O8—Cs2—O4^iii^	42.65 (9)	Si2^vii^—O2—Cs3^vii^	61.25 (14)
O5^iii^—Cs2—O4^iii^	50.93 (9)	Lu1—O2—Cs3^vii^	93.74 (12)
O7—Cs2—O4^iii^	80.39 (10)	Cs2^xiv^—O2—Cs3^vii^	126.68 (13)
Si3—Cs2—O4^iii^	53.29 (7)	Cs1^xiv^—O2—Cs3^vii^	148.21 (12)
Si2—Cs2—O4^iii^	24.01 (6)	Cs1^xii^—O2—Cs3^vii^	50.31 (5)
Si1^iii^—Cs2—O4^iii^	76.35 (6)	Si2^vii^—O2—Cs3^ix^	144.7 (2)
O3^vii^—Cs2—O4^iii^	45.57 (9)	Lu1—O2—Cs3^ix^	64.56 (10)
Cs1—Cs2—O4^iii^	84.84 (6)	Cs2^xiv^—O2—Cs3^ix^	53.55 (7)
O1—Cs2—O4^iii^	135.67 (8)	Cs1^xiv^—O2—Cs3^ix^	113.05 (10)
Cs3^viii^—Cs2—O4^iii^	110.60 (6)	Cs1^xii^—O2—Cs3^ix^	54.90 (6)
Cs3—Cs2—O4^iii^	90.87 (6)	Cs3^vii^—O2—Cs3^ix^	98.65 (9)
Si2^v^—Cs2—O4^iii^	135.59 (7)	Si2^vii^—O2—Cs3^viii^	44.36 (14)
O2^ii^—Cs2—O4^i^	40.92 (10)	Lu1—O2—Cs3^viii^	117.77 (15)
O9^iii^—Cs2—O4^i^	96.37 (10)	Cs2^xiv^—O2—Cs3^viii^	113.50 (11)
O6—Cs2—O4^i^	79.08 (9)	Cs1^xiv^—O2—Cs3^viii^	51.75 (7)
O8—Cs2—O4^i^	121.40 (9)	Cs1^xii^—O2—Cs3^viii^	136.66 (10)
O5^iii^—Cs2—O4^i^	115.22 (9)	Cs3^vii^—O2—Cs3^viii^	96.80 (7)
O7—Cs2—O4^i^	143.93 (9)	Cs3^ix^—O2—Cs3^viii^	164.19 (9)
Si3—Cs2—O4^i^	147.41 (6)	Si2^vii^—O2—Cs2^vii^	15.48 (12)
Si2—Cs2—O4^i^	105.88 (6)	Lu1—O2—Cs2^vii^	129.20 (13)
Si1^iii^—Cs2—O4^i^	107.58 (6)	Cs2^xiv^—O2—Cs2^vii^	120.81 (11)
O3^vii^—Cs2—O4^i^	159.99 (9)	Cs1^xiv^—O2—Cs2^vii^	106.26 (10)
Cs1—Cs2—O4^i^	43.99 (6)	Cs1^xii^—O2—Cs2^vii^	81.19 (8)
O1—Cs2—O4^i^	66.98 (8)	Cs3^vii^—O2—Cs2^vii^	45.79 (4)
Cs3^viii^—Cs2—O4^i^	127.87 (6)	Cs3^ix^—O2—Cs2^vii^	135.98 (10)
Cs3—Cs2—O4^i^	78.05 (6)	Cs3^viii^—O2—Cs2^vii^	56.35 (4)
Si2^v^—Cs2—O4^i^	22.85 (6)	Si2^vii^—O2—Cs3	109.84 (18)
O4^iii^—Cs2—O4^i^	114.71 (8)	Lu1—O2—Cs3	35.50 (8)
O2^ii^—Cs2—Cs1^xiv^	56.67 (9)	Cs2^xiv^—O2—Cs3	131.18 (11)
O9^iii^—Cs2—Cs1^xiv^	79.73 (7)	Cs1^xiv^—O2—Cs3	79.05 (9)
O6—Cs2—Cs1^xiv^	145.22 (8)	Cs1^xii^—O2—Cs3	121.43 (10)
O8—Cs2—Cs1^xiv^	127.74 (7)	Cs3^vii^—O2—Cs3	93.90 (7)
O5^iii^—Cs2—Cs1^xiv^	128.48 (8)	Cs3^ix^—O2—Cs3	99.66 (7)
O7—Cs2—Cs1^xiv^	85.99 (7)	Cs3^viii^—O2—Cs3	82.57 (7)
Si3—Cs2—Cs1^xiv^	113.08 (3)	Cs2^vii^—O2—Cs3	106.64 (7)
Si2—Cs2—Cs1^xiv^	152.34 (3)	Si2^vii^—O2—Cs2^x^	94.90 (16)
Si1^iii^—Cs2—Cs1^xiv^	104.07 (3)	Lu1—O2—Cs2^x^	50.45 (8)
O3^vii^—Cs2—Cs1^xiv^	120.44 (7)	Cs2^xiv^—O2—Cs2^x^	139.86 (13)
Cs1—Cs2—Cs1^xiv^	108.832 (10)	Cs1^xiv^—O2—Cs2^x^	131.88 (12)
O1—Cs2—Cs1^xiv^	44.06 (6)	Cs1^xii^—O2—Cs2^x^	71.28 (7)
Cs3^viii^—Cs2—Cs1^xiv^	59.406 (10)	Cs3^vii^—O2—Cs2^x^	44.37 (4)
Cs3—Cs2—Cs1^xiv^	88.161 (13)	Cs3^ix^—O2—Cs2^x^	86.90 (7)
Si2^v^—Cs2—Cs1^xiv^	58.63 (2)	Cs3^viii^—O2—Cs2^x^	106.64 (7)
O4^iii^—Cs2—Cs1^xiv^	165.72 (6)	Cs2^vii^—O2—Cs2^x^	81.15 (5)
O4^i^—Cs2—Cs1^xiv^	79.01 (6)	Cs3—O2—Cs2^x^	53.91 (4)
O2^ii^—Cs2—Lu1^ii^	29.98 (8)	Si3^iv^—O3—Lu1^vi^	139.2 (3)
O9^iii^—Cs2—Lu1^ii^	30.64 (8)	Si3^iv^—O3—Cs1	103.2 (2)
O6—Cs2—Lu1^ii^	131.47 (9)	Lu1^vi^—O3—Cs1	117.44 (16)
O8—Cs2—Lu1^ii^	165.07 (7)	Si3^iv^—O3—Cs3^i^	89.78 (19)
O5^iii^—Cs2—Lu1^ii^	76.86 (7)	Lu1^vi^—O3—Cs3^i^	100.59 (15)
O7—Cs2—Lu1^ii^	130.90 (7)	Cs1—O3—Cs3^i^	79.85 (11)
Si3—Cs2—Lu1^ii^	143.66 (3)	Si3^iv^—O3—Cs2^iv^	73.28 (16)
Si2—Cs2—Lu1^ii^	145.25 (3)	Lu1^vi^—O3—Cs2^iv^	89.12 (14)
Si1^iii^—Cs2—Lu1^ii^	53.07 (2)	Cs1—O3—Cs2^iv^	109.09 (13)
O3^vii^—Cs2—Lu1^ii^	122.46 (7)	Cs3^i^—O3—Cs2^iv^	162.11 (13)
Cs1—Cs2—Lu1^ii^	59.975 (10)	Si3^iv^—O3—Cs3^iii^	98.58 (18)
O1—Cs2—Lu1^ii^	97.88 (6)	Lu1^vi^—O3—Cs3^iii^	101.10 (14)
Cs3^viii^—Cs2—Lu1^ii^	64.629 (10)	Cs1—O3—Cs3^iii^	55.28 (8)
Cs3—Cs2—Lu1^ii^	138.708 (14)	Cs3^i^—O3—Cs3^iii^	135.11 (12)
Si2^v^—Cs2—Lu1^ii^	52.22 (2)	Cs2^iv^—O3—Cs3^iii^	55.62 (6)
O4^iii^—Cs2—Lu1^ii^	124.89 (6)	Si3^iv^—O3—Cs2^ii^	143.6 (2)
O4^i^—Cs2—Lu1^ii^	68.93 (6)	Lu1^vi^—O3—Cs2^ii^	66.29 (10)
Cs1^xiv^—Cs2—Lu1^ii^	62.263 (9)	Cs1—O3—Cs2^ii^	63.70 (8)
O2^ii^—Cs2—O8^v^	38.65 (10)	Cs3^i^—O3—Cs2^ii^	55.48 (6)
O9^iii^—Cs2—O8^v^	95.23 (9)	Cs2^iv^—O3—Cs2^ii^	142.24 (12)
O6—Cs2—O8^v^	108.13 (9)	Cs3^iii^—O3—Cs2^ii^	99.72 (8)
O8—Cs2—O8^v^	129.66 (6)	Si3^iv^—O3—Cs2^i^	68.55 (16)
O5^iii^—Cs2—O8^v^	139.20 (9)	Lu1^vi^—O3—Cs2^i^	86.51 (12)
O7—Cs2—O8^v^	115.96 (9)	Cs1—O3—Cs2^i^	131.12 (14)
Si3—Cs2—O8^v^	138.41 (6)	Cs3^i^—O3—Cs2^i^	52.98 (6)
Si2—Cs2—O8^v^	131.61 (6)	Cs2^iv^—O3—Cs2^i^	113.46 (10)
Si1^iii^—Cs2—O8^v^	118.10 (6)	Cs3^iii^—O3—Cs2^i^	166.07 (10)
O3^vii^—Cs2—O8^v^	159.22 (8)	Cs2^ii^—O3—Cs2^i^	94.02 (8)
Cs1—Cs2—O8^v^	76.81 (5)	Si3^iv^—O3—Cs1^xi^	18.67 (12)
O1—Cs2—O8^v^	38.53 (8)	Lu1^vi^—O3—Cs1^xi^	124.64 (15)
Cs3^viii^—Cs2—O8^v^	99.68 (5)	Cs1—O3—Cs1^xi^	117.25 (11)
Cs3—Cs2—O8^v^	73.48 (5)	Cs3^i^—O3—Cs1^xi^	80.99 (8)
Si2^v^—Cs2—O8^v^	23.12 (6)	Cs2^iv^—O3—Cs1^xi^	81.13 (7)
O4^iii^—Cs2—O8^v^	149.51 (8)	Cs3^iii^—O3—Cs1^xi^	116.34 (9)
O4^i^—Cs2—O8^v^	37.39 (8)	Cs2^ii^—O3—Cs1^xi^	136.22 (10)
Cs1^xiv^—Cs2—O8^v^	42.75 (5)	Cs2^i^—O3—Cs1^xi^	50.34 (4)
Lu1^ii^—Cs2—O8^v^	65.24 (5)	Si3^iv^—O3—Cs3^vi^	110.12 (19)
O2^ii^—Cs2—O9^iv^	89.10 (10)	Lu1^vi^—O3—Cs3^vi^	32.72 (8)
O9^iii^—Cs2—O9^iv^	138.56 (12)	Cs1—O3—Cs3^vi^	138.99 (13)
O6—Cs2—O9^iv^	38.75 (9)	Cs3^i^—O3—Cs3^vi^	122.52 (11)
O8—Cs2—O9^iv^	73.20 (9)	Cs2^iv^—O3—Cs3^vi^	60.87 (6)
O5^iii^—Cs2—O9^iv^	118.34 (10)	Cs3^iii^—O3—Cs3^vi^	95.80 (8)
O7—Cs2—O9^iv^	108.15 (9)	Cs2^ii^—O3—Cs3^vi^	99.02 (7)
Si3—Cs2—O9^iv^	99.56 (6)	Cs2^i^—O3—Cs3^vi^	84.26 (7)
Si2—Cs2—O9^iv^	62.44 (6)	Cs1^xi^—O3—Cs3^vi^	101.02 (7)
Si1^iii^—Cs2—O9^iv^	132.99 (6)	Si2^x^—O4—Lu1	138.2 (2)
O3^vii^—Cs2—O9^iv^	115.71 (9)	Si2^x^—O4—Cs1^ix^	95.68 (18)
Cs1—Cs2—O9^iv^	67.93 (6)	Lu1—O4—Cs1^ix^	119.96 (17)
O1—Cs2—O9^iv^	68.78 (8)	Si2^x^—O4—Cs3	119.6 (2)
Cs3^viii^—Cs2—O9^iv^	165.78 (6)	Lu1—O4—Cs3	88.09 (13)
Cs3—Cs2—O9^iv^	43.01 (5)	Cs1^ix^—O4—Cs3	82.89 (10)
Si2^v^—Cs2—O9^iv^	68.90 (6)	Si2^x^—O4—Cs2^x^	68.15 (15)
O4^iii^—Cs2—O9^iv^	80.87 (8)	Lu1—O4—Cs2^x^	84.32 (12)
O4^i^—Cs2—O9^iv^	48.22 (8)	Cs1^ix^—O4—Cs2^x^	153.06 (14)
Cs1^xiv^—Cs2—O9^iv^	107.67 (6)	Cs3—O4—Cs2^x^	86.77 (10)
Lu1^ii^—Cs2—O9^iv^	116.42 (6)	Si2^x^—O4—Cs2^ix^	71.29 (16)
O8^v^—Cs2—O9^iv^	69.78 (8)	Lu1—O4—Cs2^ix^	104.76 (15)
O2^ii^—Cs2—Si1^iv^	101.18 (9)	Cs1^ix^—O4—Cs2^ix^	62.36 (8)
O9^iii^—Cs2—Si1^iv^	132.27 (8)	Cs3—O4—Cs2^ix^	144.96 (13)
O6—Cs2—Si1^iv^	18.14 (7)	Cs2^x^—O4—Cs2^ix^	126.30 (10)
O8—Cs2—Si1^iv^	62.24 (7)	Si2^x^—O4—Cs3^ix^	95.57 (17)
O5^iii^—Cs2—Si1^iv^	98.14 (8)	Lu1—O4—Cs3^ix^	59.22 (10)
O7—Cs2—Si1^iv^	106.69 (7)	Cs1^ix^—O4—Cs3^ix^	100.48 (11)
Si3—Cs2—Si1^iv^	89.40 (3)	Cs3—O4—Cs3^ix^	144.32 (12)
Si2—Cs2—Si1^iv^	44.31 (3)	Cs2^x^—O4—Cs3^ix^	102.39 (8)
Si1^iii^—Cs2—Si1^iv^	117.07 (4)	Cs2^ix^—O4—Cs3^ix^	48.73 (5)
O3^vii^—Cs2—Si1^iv^	99.23 (7)	Si2^x^—O4—Cs1^x^	86.04 (18)
Cs1—Cs2—Si1^iv^	64.09 (2)	Lu1—O4—Cs1^x^	97.52 (13)
O1—Cs2—Si1^iv^	89.26 (7)	Cs1^ix^—O4—Cs1^x^	113.68 (11)
Cs3^viii^—Cs2—Si1^iv^	169.99 (2)	Cs3—O4—Cs1^x^	43.21 (6)
Cs3—Cs2—Si1^iv^	54.81 (2)	Cs2^x^—O4—Cs1^x^	46.48 (5)
Si2^v^—Cs2—Si1^iv^	84.51 (3)	Cs2^ix^—O4—Cs1^x^	155.91 (10)
O4^iii^—Cs2—Si1^iv^	59.82 (6)	Cs3^ix^—O4—Cs1^x^	145.50 (9)
O4^i^—Cs2—Si1^iv^	61.86 (6)	Si2^x^—O4—Cs1^xv^	29.68 (13)
Cs1^xiv^—Cs2—Si1^iv^	129.33 (2)	Lu1—O4—Cs1^xv^	115.56 (13)
Lu1^ii^—Cs2—Si1^iv^	122.36 (2)	Cs1^ix^—O4—Cs1^xv^	100.12 (10)
O8^v^—Cs2—Si1^iv^	90.09 (6)	Cs3—O4—Cs1^xv^	149.06 (12)
O9^iv^—Cs2—Si1^iv^	21.67 (6)	Cs2^x^—O4—Cs1^xv^	76.74 (6)
O2^ii^—Cs2—Lu1^iii^	130.86 (8)	Cs2^ix^—O4—Cs1^xv^	51.25 (5)
O9^iii^—Cs2—Lu1^iii^	76.58 (8)	Cs3^ix^—O4—Cs1^xv^	65.89 (5)
O6—Cs2—Lu1^iii^	73.00 (7)	Cs1^x^—O4—Cs1^xv^	110.48 (8)
O8—Cs2—Lu1^iii^	59.44 (7)	Si2^x^—O4—Cs2^xi^	87.39 (16)
O5^iii^—Cs2—Lu1^iii^	30.21 (7)	Lu1—O4—Cs2^xi^	130.98 (14)
O7—Cs2—Lu1^iii^	74.19 (7)	Cs1^ix^—O4—Cs2^xi^	51.96 (6)
Si3—Cs2—Lu1^iii^	52.40 (2)	Cs3—O4—Cs2^xi^	45.55 (5)
Si2—Cs2—Lu1^iii^	52.72 (2)	Cs2^x^—O4—Cs2^xi^	104.14 (9)
Si1^iii^—Cs2—Lu1^iii^	52.58 (2)	Cs2^ix^—O4—Cs2^xi^	107.65 (8)
O3^vii^—Cs2—Lu1^iii^	30.64 (6)	Cs3^ix^—O4—Cs2^xi^	152.42 (9)
Cs1—Cs2—Lu1^iii^	93.689 (13)	Cs1^x^—O4—Cs2^xi^	61.98 (5)
O1—Cs2—Lu1^iii^	152.28 (6)	Cs1^xv^—O4—Cs2^xi^	113.36 (7)
Cs3^viii^—Cs2—Lu1^iii^	80.477 (11)	Si1—O5—Lu1	137.8 (3)
Cs3—Cs2—Lu1^iii^	115.287 (12)	Si1—O5—Cs3	122.8 (2)
Si2^v^—Cs2—Lu1^iii^	146.38 (3)	Lu1—O5—Cs3	88.15 (13)
O4^iii^—Cs2—Lu1^iii^	30.99 (6)	Si1—O5—Cs2^x^	96.62 (19)
O4^i^—Cs2—Lu1^iii^	135.44 (6)	Lu1—O5—Cs2^x^	104.55 (16)
Cs1^xiv^—Cs2—Lu1^iii^	139.705 (14)	Cs3—O5—Cs2^x^	102.11 (13)
Lu1^ii^—Cs2—Lu1^iii^	105.654 (11)	Si1—O5—Cs1^x^	85.58 (16)
O8^v^—Cs2—Lu1^iii^	169.19 (5)	Lu1—O5—Cs1^x^	136.62 (16)
O9^iv^—Cs2—Lu1^iii^	111.71 (6)	Cs3—O5—Cs1^x^	59.09 (7)
Si1^iv^—Cs2—Lu1^iii^	90.34 (2)	Cs2^x^—O5—Cs1^x^	61.39 (7)
O2^ii^—Cs2—Si1	127.90 (9)	Si1—O5—Cs3^vii^	59.13 (14)
O9^iii^—Cs2—Si1	118.19 (8)	Lu1—O5—Cs3^vii^	101.90 (14)
O6—Cs2—Si1	102.32 (9)	Cs3—O5—Cs3^vii^	160.28 (14)
O8—Cs2—Si1	58.07 (7)	Cs2^x^—O5—Cs3^vii^	59.13 (7)
O5^iii^—Cs2—Si1	116.48 (8)	Cs1^x^—O5—Cs3^vii^	103.28 (9)
O7—Cs2—Si1	17.58 (8)	Si1—O5—Cs2	77.40 (17)
Si3—Cs2—Si1	43.03 (3)	Lu1—O5—Cs2	101.36 (14)
Si2—Cs2—Si1	85.06 (3)	Cs3—O5—Cs2	57.86 (7)
Si1^iii^—Cs2—Si1	120.19 (3)	Cs2^x^—O5—Cs2	146.59 (12)
O3^vii^—Cs2—Si1	61.12 (7)	Cs1^x^—O5—Cs2	85.27 (8)
Cs1—Cs2—Si1	170.18 (2)	Cs3^vii^—O5—Cs2	134.35 (10)
O1—Cs2—Si1	62.13 (6)	Si1—O5—Cs1^xiv^	91.53 (18)
Cs3^viii^—Cs2—Si1	68.87 (2)	Lu1—O5—Cs1^xiv^	57.94 (10)
Cs3—Cs2—Si1	59.79 (2)	Cs3—O5—Cs1^xiv^	89.39 (10)
Si2^v^—Cs2—Si1	121.47 (3)	Cs2^x^—O5—Cs1^xiv^	159.11 (13)
O4^iii^—Cs2—Si1	95.74 (6)	Cs1^x^—O5—Cs1^xiv^	138.73 (11)
O4^i^—Cs2—Si1	128.22 (6)	Cs3^vii^—O5—Cs1^xiv^	110.33 (9)
Cs1^xiv^—Cs2—Si1	71.51 (2)	Cs2—O5—Cs1^xiv^	54.11 (5)
Lu1^ii^—Cs2—Si1	126.07 (2)	Si1—O5—Cs1^xii^	92.82 (17)
O8^v^—Cs2—Si1	98.40 (6)	Lu1—O5—Cs1^xii^	57.75 (9)
O9^iv^—Cs2—Si1	102.45 (6)	Cs3—O5—Cs1^xii^	143.79 (12)
Si1^iv^—Cs2—Si1	107.75 (3)	Cs2^x^—O5—Cs1^xii^	77.86 (9)
Lu1^iii^—Cs2—Si1	91.76 (2)	Cs1^x^—O5—Cs1^xii^	138.65 (11)
O2^ii^—Cs2—Si3^v^	60.35 (8)	Cs3^vii^—O5—Cs1^xii^	44.34 (4)
O9^iii^—Cs2—Si3^v^	116.15 (8)	Cs2—O5—Cs1^xii^	134.69 (10)
O6—Cs2—Si3^v^	96.95 (8)	Cs1^xiv^—O5—Cs1^xii^	82.57 (6)
O8—Cs2—Si3^v^	108.11 (7)	Si2—O6—Si1^iv^	135.8 (2)
O5^iii^—Cs2—Si3^v^	159.88 (8)	Si2—O6—Cs2	94.35 (17)
O7—Cs2—Si3^v^	99.75 (8)	Si1^iv^—O6—Cs2	125.96 (19)
Si3—Cs2—Si3^v^	118.10 (3)	Si2—O6—Cs3	97.1 (2)
Si2—Cs2—Si3^v^	115.15 (3)	Si1^iv^—O6—Cs3	83.32 (18)
Si1^iii^—Cs2—Si3^v^	139.86 (3)	Cs2—O6—Cs3	69.46 (10)
O3^vii^—Cs2—Si3^v^	142.10 (7)	Si2—O6—Cs3^iii^	86.44 (18)
Cs1—Cs2—Si3^v^	90.37 (2)	Si1^iv^—O6—Cs3^iii^	110.2 (2)
O1—Cs2—Si3^v^	20.57 (6)	Cs2—O6—Cs3^iii^	87.13 (9)
Cs3^viii^—Cs2—Si3^v^	107.82 (2)	Cs3—O6—Cs3^iii^	156.51 (11)
Cs3—Cs2—Si3^v^	52.07 (2)	Si2—O6—Cs1	132.60 (19)
Si2^v^—Cs2—Si3^v^	42.05 (3)	Si1^iv^—O6—Cs1	86.99 (16)
O4^iii^—Cs2—Si3^v^	137.98 (6)	Cs2—O6—Cs1	61.75 (6)
O4^i^—Cs2—Si3^v^	46.50 (6)	Cs3—O6—Cs1	109.44 (10)
Cs1^xiv^—Cs2—Si3^v^	48.76 (2)	Cs3^iii^—O6—Cs1	54.45 (6)
Lu1^ii^—Cs2—Si3^v^	86.82 (2)	Si2—O6—Cs1^xiii^	49.41 (14)
O8^v^—Cs2—Si3^v^	21.86 (6)	Si1^iv^—O6—Cs1^xiii^	104.59 (18)
O9^iv^—Cs2—Si3^v^	58.92 (6)	Cs2—O6—Cs1^xiii^	126.75 (11)
Si1^iv^—Cs2—Si3^v^	80.59 (3)	Cs3—O6—Cs1^xiii^	139.09 (11)
Lu1^iii^—Cs2—Si3^v^	167.27 (2)	Cs3^iii^—O6—Cs1^xiii^	57.90 (7)
Si1—Cs2—Si3^v^	82.63 (3)	Cs1—O6—Cs1^xiii^	111.00 (11)
O1—Cs3—O9^iv^	100.78 (11)	Si2—O6—Cs1^x^	51.55 (15)
O1—Cs3—O5	63.24 (11)	Si1^iv^—O6—Cs1^x^	104.07 (18)
O9^iv^—Cs3—O5	154.42 (12)	Cs2—O6—Cs1^x^	92.17 (11)
O1—Cs3—O4	63.56 (11)	Cs3—O6—Cs1^x^	49.00 (6)
O9^iv^—Cs3—O4	131.81 (11)	Cs3^iii^—O6—Cs1^x^	137.84 (9)
O5—Cs3—O4	61.42 (11)	Cs1—O6—Cs1^x^	152.70 (12)
O1—Cs3—O3^ix^	50.44 (10)	Cs1^xiii^—O6—Cs1^x^	90.58 (7)
O9^iv^—Cs3—O3^ix^	58.46 (11)	Si2—O6—Cs2^iv^	103.2 (2)
O5—Cs3—O3^ix^	112.92 (10)	Si1^iv^—O6—Cs2^iv^	64.81 (17)
O4—Cs3—O3^ix^	80.24 (11)	Cs2—O6—Cs2^iv^	132.16 (13)
O1—Cs3—O8	111.75 (11)	Cs3—O6—Cs2^iv^	147.93 (10)
O9^iv^—Cs3—O8	89.80 (10)	Cs3^iii^—O6—Cs2^iv^	50.83 (6)
O5—Cs3—O8	79.16 (10)	Cs1—O6—Cs2^iv^	73.98 (8)
O4—Cs3—O8	138.25 (10)	Cs1^xiii^—O6—Cs2^iv^	53.83 (6)
O3^ix^—Cs3—O8	131.18 (10)	Cs1^x^—O6—Cs2^iv^	133.32 (8)
O1—Cs3—O6	111.83 (10)	Si2—O6—Cs2^xi^	113.05 (18)
O9^iv^—Cs3—O6	46.36 (10)	Si1^iv^—O6—Cs2^xi^	37.54 (14)
O5—Cs3—O6	118.27 (10)	Cs2—O6—Cs2^xi^	115.84 (12)
O4—Cs3—O6	175.22 (10)	Cs3—O6—Cs2^xi^	51.19 (5)
O3^ix^—Cs3—O6	95.85 (9)	Cs3^iii^—O6—Cs2^xi^	147.15 (11)
O8—Cs3—O6	43.48 (9)	Cs1—O6—Cs2^xi^	114.21 (8)
O1—Cs3—Cs1^x^	137.68 (8)	Cs1^xiii^—O6—Cs2^xi^	114.35 (8)
O9^iv^—Cs3—Cs1^x^	116.05 (8)	Cs1^x^—O6—Cs2^xi^	68.09 (6)
O5—Cs3—Cs1^x^	74.79 (8)	Cs2^iv^—O6—Cs2^xi^	97.58 (7)
O4—Cs3—Cs1^x^	101.55 (8)	Si2—O6—Cs3^iv^	105.48 (16)
O3^ix^—Cs3—Cs1^x^	171.62 (7)	Si1^iv^—O6—Cs3^iv^	35.56 (12)
O8—Cs3—Cs1^x^	51.87 (7)	Cs2—O6—Cs3^iv^	160.16 (11)
O6—Cs3—Cs1^x^	82.71 (6)	Cs3—O6—Cs3^iv^	106.91 (8)
O1—Cs3—Si3^v^	24.90 (8)	Cs3^iii^—O6—Cs3^iv^	94.37 (9)
O9^iv^—Cs3—Si3^v^	78.98 (8)	Cs1—O6—Cs3^iv^	103.37 (9)
O5—Cs3—Si3^v^	87.92 (8)	Cs1^xiii^—O6—Cs3^iv^	69.30 (6)
O4—Cs3—Si3^v^	71.10 (9)	Cs1^x^—O6—Cs3^iv^	99.77 (7)
O3^ix^—Cs3—Si3^v^	25.59 (7)	Cs2^iv^—O6—Cs3^iv^	43.78 (4)
O8—Cs3—Si3^v^	123.47 (7)	Cs2^xi^—O6—Cs3^iv^	55.96 (4)
O6—Cs3—Si3^v^	104.20 (7)	Si2—O6—Cs2^xiii^	17.22 (10)
Cs1^x^—Cs3—Si3^v^	162.58 (3)	Si1^iv^—O6—Cs2^xiii^	118.63 (16)
O1—Cs3—Lu1	37.14 (8)	Cs2—O6—Cs2^xiii^	110.49 (10)
O9^iv^—Cs3—Lu1	136.42 (8)	Cs3—O6—Cs2^xiii^	96.83 (9)
O5—Cs3—Lu1	36.96 (7)	Cs3^iii^—O6—Cs2^xiii^	93.24 (8)
O4—Cs3—Lu1	36.76 (7)	Cs1—O6—Cs2^xiii^	145.60 (10)
O3^ix^—Cs3—Lu1	79.34 (7)	Cs1^xiii^—O6—Cs2^xiii^	43.78 (3)
O8—Cs3—Lu1	113.09 (7)	Cs1^x^—O6—Cs2^xiii^	47.91 (4)
O6—Cs3—Lu1	140.02 (6)	Cs2^iv^—O6—Cs2^xiii^	95.49 (7)
Cs1^x^—Cs3—Lu1	107.062 (14)	Cs2^xi^—O6—Cs2^xiii^	99.44 (7)
Si3^v^—Cs3—Lu1	57.44 (2)	Cs3^iv^—O6—Cs2^xiii^	89.20 (6)
O1—Cs3—Si1^iv^	116.51 (8)	Si3—O7—Si1	133.0 (3)
O9^iv^—Cs3—Si1^iv^	23.97 (8)	Si3—O7—Cs2	90.35 (17)
O5—Cs3—Si1^iv^	144.07 (9)	Si1—O7—Cs2	126.6 (2)
O4—Cs3—Si1^iv^	153.85 (8)	Si3—O7—Cs1^vii^	79.85 (16)
O3^ix^—Cs3—Si1^iv^	81.49 (7)	Si1—O7—Cs1^vii^	109.26 (19)
O8—Cs3—Si1^iv^	67.60 (7)	Cs2—O7—Cs1^vii^	107.70 (11)
O6—Cs3—Si1^iv^	25.86 (6)	Si3—O7—Cs3^viii^	116.20 (18)
Cs1^x^—Cs3—Si1^iv^	94.03 (2)	Si1—O7—Cs3^viii^	106.73 (17)
Si3^v^—Cs3—Si1^iv^	98.92 (3)	Cs2—O7—Cs3^viii^	64.09 (7)
Lu1—Cs3—Si1^iv^	153.38 (3)	Cs1^vii^—O7—Cs3^viii^	58.27 (6)
O1—Cs3—Cs2^xi^	127.70 (8)	Si3—O7—Cs3	92.68 (16)
O9^iv^—Cs3—Cs2^xi^	52.01 (9)	Si1—O7—Cs3	82.41 (16)
O5—Cs3—Cs2^xi^	153.55 (9)	Cs2—O7—Cs3	63.04 (7)
O4—Cs3—Cs2^xi^	99.79 (7)	Cs1^vii^—O7—Cs3	168.32 (12)
O3^ix^—Cs3—Cs2^xi^	78.98 (7)	Cs3^viii^—O7—Cs3	118.79 (10)
O8—Cs3—Cs2^xi^	111.58 (7)	Si3—O7—Cs1^x^	58.58 (13)
O6—Cs3—Cs2^xi^	82.05 (6)	Si1—O7—Cs1^x^	82.85 (15)
Cs1^x^—Cs3—Cs2^xi^	92.647 (13)	Cs2—O7—Cs1^x^	103.29 (10)
Si3^v^—Cs3—Cs2^xi^	104.05 (3)	Cs1^vii^—O7—Cs1^x^	127.58 (11)
Lu1—Cs3—Cs2^xi^	134.225 (14)	Cs3^viii^—O7—Cs1^x^	167.06 (12)
Si1^iv^—Cs3—Cs2^xi^	58.23 (2)	Cs3—O7—Cs1^x^	52.75 (5)
O1—Cs3—Cs2	67.00 (8)	Si3—O7—Cs2^vii^	121.83 (18)
O9^iv^—Cs3—Cs2	75.21 (9)	Si1—O7—Cs2^vii^	60.47 (14)
O5—Cs3—Cs2	79.94 (9)	Cs2—O7—Cs2^vii^	128.36 (10)
O4—Cs3—Cs2	126.81 (8)	Cs1^vii^—O7—Cs2^vii^	50.48 (5)
O3^ix^—Cs3—Cs2	83.30 (8)	Cs3^viii^—O7—Cs2^vii^	65.49 (6)
O8—Cs3—Cs2	51.14 (7)	Cs3—O7—Cs2^vii^	140.49 (10)
O6—Cs3—Cs2	49.52 (6)	Cs1^x^—O7—Cs2^vii^	127.45 (9)
Cs1^x^—Cs3—Cs2	101.762 (14)	Si3—O7—Cs3^xiii^	33.75 (12)
Si3^v^—Cs3—Cs2	72.51 (2)	Si1—O7—Cs3^xiii^	116.53 (18)
Lu1—Cs3—Cs2	90.584 (12)	Cs2—O7—Cs3^xiii^	116.31 (10)
Si1^iv^—Cs3—Cs2	68.89 (2)	Cs1^vii^—O7—Cs3^xiii^	53.19 (6)
Cs2^xi^—Cs3—Cs2	125.955 (16)	Cs3^viii^—O7—Cs3^xiii^	106.82 (9)
O1—Cs3—O6^x^	106.20 (10)	Cs3—O7—Cs3^xiii^	122.78 (9)
O9^iv^—Cs3—O6^x^	129.89 (11)	Cs1^x^—O7—Cs3^xiii^	75.41 (6)
O5—Cs3—O6^x^	75.42 (11)	Cs2^vii^—O7—Cs3^xiii^	88.17 (7)
O4—Cs3—O6^x^	43.01 (10)	Si3—O7—Cs2^x^	101.95 (16)
O3^ix^—Cs3—O6^x^	111.89 (9)	Si1—O7—Cs2^x^	34.70 (11)
O8—Cs3—O6^x^	116.91 (9)	Cs2—O7—Cs2^x^	126.23 (11)
O6—Cs3—O6^x^	141.73 (6)	Cs1^vii^—O7—Cs2^x^	125.89 (10)
Cs1^x^—Cs3—O6^x^	65.96 (6)	Cs3^viii^—O7—Cs2^x^	141.00 (10)
Si3^v^—Cs3—O6^x^	112.22 (6)	Cs3—O7—Cs2^x^	64.24 (6)
Lu1—Cs3—O6^x^	73.34 (6)	Cs1^x^—O7—Cs2^x^	48.17 (4)
Si1^iv^—Cs3—O6^x^	131.56 (7)	Cs2^vii^—O7—Cs2^x^	88.32 (7)
Cs2^xi^—Cs3—O6^x^	78.22 (6)	Cs3^xiii^—O7—Cs2^x^	100.28 (7)
Cs2—Cs3—O6^x^	154.60 (6)	Si3—O7—Cs1^xiv^	145.31 (19)
O1—Cs3—O7^xi^	142.13 (10)	Si1—O7—Cs1^xiv^	78.16 (15)
O9^iv^—Cs3—O7^xi^	98.13 (10)	Cs2—O7—Cs1^xiv^	55.03 (6)
O5—Cs3—O7^xi^	106.53 (10)	Cs1^vii^—O7—Cs1^xiv^	106.55 (9)
O4—Cs3—O7^xi^	79.38 (10)	Cs3^viii^—O7—Cs1^xiv^	50.44 (4)
O3^ix^—Cs3—O7^xi^	118.35 (10)	Cs3—O7—Cs1^xiv^	74.63 (6)
O8—Cs3—O7^xi^	100.78 (9)	Cs1^x^—O7—Cs1^xiv^	125.86 (9)
O6—Cs3—O7^xi^	105.00 (8)	Cs2^vii^—O7—Cs1^xiv^	84.31 (6)
Cs1^x^—Cs3—O7^xi^	54.57 (6)	Cs3^xiii^—O7—Cs1^xiv^	157.05 (9)
Si3^v^—Cs3—O7^xi^	135.48 (6)	Cs2^x^—O7—Cs1^xiv^	101.13 (7)
Lu1—Cs3—O7^xi^	112.19 (6)	Si3—O7—Cs3^vii^	122.73 (17)
Si1^iv^—Cs3—O7^xi^	93.14 (6)	Si1—O7—Cs3^vii^	21.86 (12)
Cs2^xi^—Cs3—O7^xi^	48.82 (6)	Cs2—O7—Cs3^vii^	145.54 (11)
Cs2—Cs3—O7^xi^	150.34 (6)	Cs1^vii^—O7—Cs3^vii^	89.11 (8)
O6^x^—Cs3—O7^xi^	38.86 (8)	Cs3^viii^—O7—Cs3^vii^	103.78 (8)
O1—Cs3—Cs1^ix^	69.56 (8)	Cs3—O7—Cs3^vii^	102.51 (8)
O9^iv^—Cs3—Cs1^ix^	84.40 (8)	Cs1^x^—O7—Cs3^vii^	88.34 (7)
O5—Cs3—Cs1^ix^	106.18 (8)	Cs2^vii^—O7—Cs3^vii^	44.34 (4)
O4—Cs3—Cs1^ix^	47.54 (8)	Cs3^xiii^—O7—Cs3^vii^	97.92 (7)
O3^ix^—Cs3—Cs1^ix^	44.94 (8)	Cs2^x^—O7—Cs3^vii^	44.05 (3)
O8—Cs3—Cs1^ix^	174.19 (7)	Cs1^xiv^—O7—Cs3^vii^	91.78 (6)
O6—Cs3—Cs1^ix^	130.72 (6)	Si2—O8—Si3	132.9 (2)
Cs1^x^—Cs3—Cs1^ix^	131.185 (15)	Si2—O8—Cs1^x^	110.77 (19)
Si3^v^—Cs3—Cs1^ix^	55.41 (2)	Si3—O8—Cs1^x^	93.66 (16)
Lu1—Cs3—Cs1^ix^	71.536 (10)	Si2—O8—Cs2	93.67 (16)
Si1^iv^—Cs3—Cs1^ix^	106.66 (3)	Si3—O8—Cs2	92.90 (17)
Cs2^xi^—Cs3—Cs1^ix^	64.613 (12)	Cs1^x^—O8—Cs2	138.75 (13)
Cs2—Cs3—Cs1^ix^	126.802 (14)	Si2—O8—Cs3	106.38 (17)
O6^x^—Cs3—Cs1^ix^	67.30 (6)	Si3—O8—Cs3	120.04 (18)
O7^xi^—Cs3—Cs1^ix^	80.07 (6)	Cs1^x^—O8—Cs3	68.85 (8)
O1—Cs3—O7	79.30 (10)	Cs2—O8—Cs3	72.67 (8)
O9^iv^—Cs3—O7	118.83 (10)	Si2—O8—Cs2^xiii^	67.39 (13)
O5—Cs3—O7	41.93 (10)	Si3—O8—Cs2^xiii^	87.19 (15)
O4—Cs3—O7	103.31 (9)	Cs1^x^—O8—Cs2^xiii^	68.13 (7)
O3^ix^—Cs3—O7	122.38 (9)	Cs2—O8—Cs2^xiii^	152.92 (12)
O8—Cs3—O7	39.81 (9)	Cs3—O8—Cs2^xiii^	129.88 (11)
O6—Cs3—O7	76.39 (8)	Si2—O8—Cs3^xiii^	101.07 (15)
Cs1^x^—Cs3—O7	65.40 (6)	Si3—O8—Cs3^xiii^	38.67 (11)
Si3^v^—Cs3—O7	100.27 (6)	Cs1^x^—O8—Cs3^xiii^	87.23 (8)
Lu1—Cs3—O7	73.32 (6)	Cs2—O8—Cs3^xiii^	121.08 (10)
Si1^iv^—Cs3—O7	102.24 (6)	Cs3—O8—Cs3^xiii^	148.47 (11)
Cs2^xi^—Cs3—O7	150.75 (6)	Cs2^xiii^—O8—Cs3^xiii^	49.74 (4)
Cs2—Cs3—O7	48.17 (6)	Si2—O8—Cs1^xiii^	22.24 (10)
O6^x^—Cs3—O7	107.28 (8)	Si3—O8—Cs1^xiii^	114.30 (16)
O7^xi^—Cs3—O7	118.79 (10)	Cs1^x^—O8—Cs1^xiii^	104.99 (10)
Cs1^ix^—Cs3—O7	144.45 (6)	Cs2—O8—Cs1^xiii^	109.11 (9)
O1—Cs3—Si2^x^	83.37 (8)	Cs3—O8—Cs1^xiii^	125.51 (10)
O9^iv^—Cs3—Si2^x^	131.40 (9)	Cs2^xiii^—O8—Cs1^xiii^	47.69 (4)
O5—Cs3—Si2^x^	69.40 (9)	Cs3^xiii^—O8—Cs1^xiii^	79.36 (6)
O4—Cs3—Si2^x^	19.82 (8)	Si2—O8—Cs1^vii^	124.34 (17)
O3^ix^—Cs3—Si2^x^	92.60 (8)	Si3—O8—Cs1^vii^	17.66 (11)
O8—Cs3—Si2^x^	133.96 (7)	Cs1^x^—O8—Cs1^vii^	111.01 (10)
O6—Cs3—Si2^x^	164.69 (6)	Cs2—O8—Cs1^vii^	77.66 (8)
Cs1^x^—Cs3—Si2^x^	87.07 (2)	Cs3—O8—Cs1^vii^	122.06 (10)
Si3^v^—Cs3—Si2^x^	88.85 (3)	Cs2^xiii^—O8—Cs1^vii^	96.71 (7)
Lu1—Cs3—Si2^x^	54.30 (2)	Cs3^xiii^—O8—Cs1^vii^	47.13 (4)
Si1^iv^—Cs3—Si2^x^	145.32 (3)	Cs1^xiii^—O8—Cs1^vii^	110.80 (7)
Cs2^xi^—Cs3—Si2^x^	87.09 (2)	Si2—O8—Cs3^iii^	36.07 (12)
Cs2—Cs3—Si2^x^	144.67 (2)	Si3—O8—Cs3^iii^	111.34 (16)
O6^x^—Cs3—Si2^x^	23.68 (6)	Cs1^x^—O8—Cs3^iii^	146.83 (11)
O7^xi^—Cs3—Si2^x^	59.69 (6)	Cs2—O8—Cs3^iii^	63.85 (6)
Cs1^ix^—Cs3—Si2^x^	51.38 (2)	Cs3—O8—Cs3^iii^	112.60 (9)
O7—Cs3—Si2^x^	109.57 (6)	Cs2^xiii^—O8—Cs3^iii^	90.91 (7)
O1—Cs3—Si2	121.10 (8)	Cs3^xiii^—O8—Cs3^iii^	98.70 (6)
O9^iv^—Cs3—Si2	68.31 (8)	Cs1^xiii^—O8—Cs3^iii^	45.48 (3)
O5—Cs3—Si2	101.67 (8)	Cs1^vii^—O8—Cs3^iii^	96.27 (6)
O4—Cs3—Si2	159.68 (8)	Si1—O9—Lu1^xiii^	137.7 (2)
O3^ix^—Cs3—Si2	118.59 (8)	Si1—O9—Cs3^vii^	107.17 (19)
O8—Cs3—Si2	22.61 (7)	Lu1^xiii^—O9—Cs3^vii^	111.71 (15)
O6—Cs3—Si2	23.29 (6)	Si1—O9—Cs2^x^	99.43 (19)
Cs1^x^—Cs3—Si2	61.02 (2)	Lu1^xiii^—O9—Cs2^x^	103.89 (15)
Si3^v^—Cs3—Si2	122.23 (3)	Cs3^vii^—O9—Cs2^x^	78.56 (10)
Lu1—Cs3—Si2	133.90 (2)	Si1—O9—Cs1^x^	87.21 (16)
Si1^iv^—Cs3—Si2	45.30 (3)	Lu1^xiii^—O9—Cs1^x^	73.42 (11)
Cs2^xi^—Cs3—Si2	91.84 (2)	Cs3^vii^—O9—Cs1^x^	140.36 (14)
Cs2—Cs3—Si2	54.15 (2)	Cs2^x^—O9—Cs1^x^	62.49 (8)
O6^x^—Cs3—Si2	125.41 (6)	Si1—O9—Cs2^vii^	82.40 (17)
O7^xi^—Cs3—Si2	96.34 (6)	Lu1^xiii^—O9—Cs2^vii^	101.61 (14)
Cs1^ix^—Cs3—Si2	151.80 (2)	Cs3^vii^—O9—Cs2^vii^	61.77 (8)
O7—Cs3—Si2	61.14 (6)	Cs2^x^—O9—Cs2^vii^	138.56 (12)
Si2^x^—Cs3—Si2	148.01 (2)	Cs1^x^—O9—Cs2^vii^	157.83 (11)
O1—Cs3—Si1	72.06 (8)	Si1—O9—Cs1^vii^	73.68 (16)
O9^iv^—Cs3—Si1	141.23 (9)	Lu1^xiii^—O9—Cs1^vii^	76.55 (11)
O5—Cs3—Si1	18.98 (8)	Cs3^vii^—O9—Cs1^vii^	112.61 (12)
O4—Cs3—Si1	80.28 (8)	Cs2^x^—O9—Cs1^vii^	168.03 (12)
O3^ix^—Cs3—Si1	122.08 (7)	Cs1^x^—O9—Cs1^vii^	106.83 (9)
O8—Cs3—Si1	60.36 (7)	Cs2^vii^—O9—Cs1^vii^	51.41 (5)
O6—Cs3—Si1	99.67 (6)	Si1—O9—Cs1^xvi^	154.9 (2)
Cs1^x^—Cs3—Si1	66.26 (2)	Lu1^xiii^—O9—Cs1^xvi^	66.01 (10)
Si3^v^—Cs3—Si1	96.60 (3)	Cs3^vii^—O9—Cs1^xvi^	56.91 (6)
Lu1—Cs3—Si1	54.03 (2)	Cs2^x^—O9—Cs1^xvi^	60.38 (7)
Si1^iv^—Cs3—Si1	125.54 (3)	Cs1^x^—O9—Cs1^xvi^	94.98 (8)
Cs2^xi^—Cs3—Si1	158.23 (2)	Cs2^vii^—O9—Cs1^xvi^	102.73 (8)
Cs2—Cs3—Si1	66.87 (2)	Cs1^vii^—O9—Cs1^xvi^	128.74 (9)
O6^x^—Cs3—Si1	87.73 (6)	Si1—O9—Cs3	45.19 (13)
O7^xi^—Cs3—Si1	110.63 (6)	Lu1^xiii^—O9—Cs3	115.62 (13)
Cs1^ix^—Cs3—Si1	124.85 (2)	Cs3^vii^—O9—Cs3	126.33 (12)
O7—Cs3—Si1	23.37 (6)	Cs2^x^—O9—Cs3	67.01 (7)
Si2^x^—Cs3—Si1	86.50 (3)	Cs1^x^—O9—Cs3	44.85 (4)
Si2—Cs3—Si1	82.77 (3)	Cs2^vii^—O9—Cs3	127.56 (9)
O1—Cs3—Lu1^i^	75.95 (8)	Cs1^vii^—O9—Cs3	101.81 (7)
O9^iv^—Cs3—Lu1^i^	28.40 (8)	Cs1^xvi^—O9—Cs3	125.13 (9)
O5—Cs3—Lu1^i^	138.58 (8)	Si1—O9—Cs3^xiii^	103.09 (17)
O4—Cs3—Lu1^i^	107.15 (8)	Lu1^xiii^—O9—Cs3^xiii^	35.53 (7)
O3^ix^—Cs3—Lu1^i^	30.07 (7)	Cs3^vii^—O9—Cs3^xiii^	135.25 (12)
O8—Cs3—Lu1^i^	111.64 (7)	Cs2^x^—O9—Cs3^xiii^	127.78 (11)
O6—Cs3—Lu1^i^	69.75 (6)	Cs1^x^—O9—Cs3^xiii^	72.23 (6)
Cs1^x^—Cs3—Lu1^i^	144.048 (14)	Cs2^vii^—O9—Cs3^xiii^	91.08 (8)
Si3^v^—Cs3—Lu1^i^	52.12 (2)	Cs1^vii^—O9—Cs3^xiii^	47.25 (4)
Lu1—Cs3—Lu1^i^	108.890 (9)	Cs1^xvi^—O9—Cs3^xiii^	101.38 (7)
Si1^iv^—Cs3—Lu1^i^	51.75 (2)	Cs3—O9—Cs3^xiii^	98.38 (7)
Cs2^xi^—Cs3—Lu1^i^	61.779 (9)		

Symmetry codes: (i) −*x*, −*y*+1, *z*+1/2; (ii) −*x*, −*y*, *z*+1/2; (iii) −*x*+1/2, *y*−1/2, *z*+1/2; (iv) −*x*+1/2, *y*+1/2, *z*+1/2; (v) *x*−1/2, −*y*+1/2, *z*; (vi) *x*, *y*, *z*+1; (vii) −*x*+1/2, *y*−1/2, *z*−1/2; (viii) *x*, *y*−1, *z*; (ix) −*x*, −*y*+1, *z*−1/2; (x) −*x*+1/2, *y*+1/2, *z*−1/2; (xi) *x*, *y*+1, *z*; (xii) *x*, *y*, *z*−1; (xiii) *x*+1/2, −*y*+1/2, *z*; (xiv) −*x*, −*y*, *z*−1/2; (xv) *x*, *y*+1, *z*−1; (xvi) *x*+1/2, −*y*+1/2, *z*−1.
